# Geographically weighted bivariate zero inflated generalized Poisson regression model and its application

**DOI:** 10.1016/j.heliyon.2021.e07491

**Published:** 2021-07-08

**Authors:** Purhadi, Dewi Novita Sari, Qurotul Aini, Irhamah

**Affiliations:** a Department of Statistics, Faculty of Science and Data Analytics, Institut Teknologi Sepuluh Nopember, Surabaya, 60111, Indonesia; b BPS-Statistics Indonesia, Jl. Dr. Sutomo 6-8, Jakarta 10710, Indonesia

**Keywords:** GWBZIGPR, MLE, MLRT, Maternal mortality rate

## Abstract

This study discusses the development of Zero Inflated Generalized Poisson Regression (ZIGPR) with two response variables, that is Bivariate ZIGPR (BZIGPR). The extension of the ZIGPR model by considering spatial factor called Geographically Weighted Zero Inflated Generalized Poisson Regression (GWBZIGPR). The GWBZIGPR produces a local parameter estimator for each location of observation. The parameter estimation using the Maximum Likelihood Estimation (MLE) method obtained an equation that did not closed-form so that the numerical iteration of Berndt Hall Hall Hausman (BHHH) is used. The data used in this study are the number of pregnant maternal mortality and postpartum maternal mortality data in 91 sub-districts in Pekalongan Residency, Central Java Province. The results showed that the Akaike Information Criterion Corrected (AICc) value in the GWBZIGPR model is smaller than BZIGPR, so it means that the GWBZIGPR is better than the BZIGPR for modeling the number of pregnant maternal mortality and postpartum maternal mortality in Pekalongan Residency. The results of this study will assist local governments in anticipating the causes of maternal mortality.

## 1 Introduction

Poisson regression is a regression analysis where the response follows the Poisson distribution. The Poisson regression model has a limitation where the variance equal to the mean, known as equidispersion. According to McCullagh and Nelder (1989), if the equidispersion assumption is not met, the standard error will be biased and the test statistics derived from the model become less precise so that less valid conclusions are obtained [1]. Therefore, the Poisson regression model cannot be applied when under/overdispersion occurs. Generalized Poisson Distribution (GPD) is an alternative model that can be used to handling under/overdispersion. The GPD has one additional parameter that makes the distribution more flexible in dealing with underdispersion or overdispersion [2].

The number of zero observations that is more than the number of zeros estimated by the model or called excess zero is also a problem besides underdispersion or overdispersion. Zero Inflated Poisson (ZIP) regression model was used to handle this problem [3]. The ZIP model is applied to biological data and shows that the ZIP model is not suitable for dealing with overdispersion cases. Overdispersion that occurs in data that has more zero causes the parameter estimation in the ZIP to be biased [4]. Therefore, Famoye and Singh (2006) suggest the use of the Zero Inflated Generalized Poisson (ZIGP) model [5]. The development of bivariate ZIGP (BZIGP) was carried out by Zhang and Huang (2015) [6].

The parameters generated by the BZIGP regression are considered suitable for all observation locations. [7] and [8] developed a spatial model known as Geographically Weighted Regression (GWR). GWR is spatial modeling with a local point approach so that the resulting model parameters are local for each point or location where the data is observed. Research on the GWR model has been conducted by [9] and [10]. [9] discusses Geographically Weighted Poisson Regression (GWPR) which is the development of Poisson regression with regard to spatial factors while [10] conducted research on the GWR model with a bivariate response. Maternal mortality rate is count data where the number of events does not have an upper limit and is always a non-negative integer. [11] states that the distribution for such count data is the Poisson distribution. The number of pregnant maternal and postpartum deaths in Pekalongan Residency in 2017 are data with overdispersion and contains excess zero so that this study will examine the parameter estimation and hypothesis testing of Geographically Weighted Bivariate Zero Inflated Generalized Poisson Regression in cases of the number of pregnant maternal and postpartum deaths at Pekalongan Residency in Central Java in 2017. The results of the study are expected to determine the factors that have a significant effect on the number of pregnant maternal and postpartum deaths in Pekalongan Residency, Central Java Province in 2017. Thus the result of this study will assist local governments in anticipating the causes of maternal mortality.

The discussion in this study is divided into several sections. In section 2, we discuss about material and method. In section 3, we discuss parameter estimation using MLE and hypothesis testing for GWBZIGPR using MLRT. In section 4 we discuss the factors that significantly influence the number of pregnant maternal mortality and postpartum maternal mortality in Pekalongan Residency in 2017 and its interpretation of the model. Section 5 provides a conclusion. The scope of the study is restricted to 91 sub-districts in Pekalongan Residency. BHHH iteration will be used if the parameter estimation results do not yield a close-form solution.

## 2 Material and method

### 2.1 Source of data

This study uses secondary data from the Central Java Provincial Health Office in 2017. The observation units are 91 sub-districts in Pekalongan Residency, Central Java Province. Table 1 shows that the variables used in this study consisted of 2 response variables and 7 predictor variables.

**Table 1:** Research variable.

Variable	Information
Y_1_	The number of pregnant maternal mortality
Y_2_	The number of postpartum maternal mortality
X_1_	The percentage of K1 visits by pregnant women (K1 visits are antenatal care visits at least once in the first trimester until 14 weeks of gestation)
X_2_	The percentage of K4 visits by pregnant women (K4 visits are antenatal care visits at least twice in the third trimester at 28 to 36 weeks of gestation)
X_3_	The percentage of childbirth assisted by health workers
X_4_	The percentage of TT2 + immunization
X_5_	The percentage of pregnant women who received Fe3 tablet
X_6_	The percentage of pregnant women with obstetric complications who are treated
X_7_	The ratio of midwives per 100,000 population

### 2.2 ZIGPR

The probability density function of the ZIGP is [5]:

(1) $$P\left(Y_{i}=y_{i}\right)=\left\{ \begin{array}{c} p_{i}+\left(1-p_{i}\right)\exp\left(\frac{-\mu_{i}}{1+\varphi \mu_{i}}\right);\;y_{i}=0\\ \left(1-p_{i}\right){\left(\frac{\mu_{i}}{1+\varphi \mu_{i}}\right)}^{y_{i}}\frac{{\left(1+\varphi y_{i}\right)}^{y_{i}-1}}{y_{i}!}\exp\left(\frac{-\mu_{i}\left(1+\varphi y_{i}\right)}{1+\varphi \mu_{i}}\right);\;y_{i}>0 \end{array}\right.$$

ZIGPR model: $\mu_{i}=\exp\left(x_{i}^{T}\beta \right)$, φ=dispersion parameter,

$$p_{i}=\frac{\exp\left(x_{i}^{T}\gamma \right)}{1+\exp\left(x_{i}^{T}\gamma \right)}\text{ and }\left(1-p_{i}\right)=\frac{1}{1+\exp\left(x_{i}^{T}\gamma \right)}$$

### 2.3 BZIGPR

The probability density function of the BZIGP distribution is [6] $\left(Y_{1i},Y_{2i}\right)∼\mathrm{ZIGP}\left(\gamma_{1},\gamma_{2},\beta_{1},\beta_{2},\varphi_{1},\varphi_{2},\eta \right)$ for i=1,...,n and $Y_{\mathrm{obs}}=\left\{\left(y_{1i},y_{2i}\right):i=1,...,n\right\}$. Defined:

$$I_{1}=\left\{i:y_{1i}=0,\;y_{2i}=0,\;i=1,2,...,n\right\},\;n_{1}=\#\left\{I_{1}\right\},I_{2}=\left\{i:y_{1i}=0,\;y_{2i}>0,\;i=1,2,...,n\right\},\;n_{2}=\#\left\{I_{2}\right\},I_{3}=\left\{i:y_{1i}>0,\;y_{2i}=0,\;i=1,2,...,n\right\},\;n_{3}=\#\left\{I_{3}\right\},I_{4}=\left\{i:y_{1i}>0,\;y_{2i}>0,\;i=1,2,...,n\right\},\;n_{4}=\#\left\{I_{4}\right\}n_{4}=n-n_{1}-n_{2}-n_{3}$$

BZIGPR model: $\mu_{li}=\exp\left(x_{i}^{T}\beta_{l}\right)$, $p_{li}=\frac{\exp\left(x_{i}^{T}\gamma_{l}\right)}{1+\exp\left(x_{i}^{T}\gamma_{l}\right)}$ and $\left(1-p_{li}\right)=\frac{1}{1+\exp\left(x_{i}^{T}\gamma_{l}\right)}$, l=1,2; i=1,2,...,n. So that the pdf of BZIGPR is defined

$$f\left(y_{1i},y_{2i}\right)=\left\{ \begin{array}{c} A_{i},\left(\text{if}\;y_{1i}=0\;\text{and}\;y_{2i}=0\right)\\ B_{i},\left(\text{if}\;y_{1i}=0\;\text{and}\;y_{2i}>0\right)\\ C_{i},\left(\text{if}\;y_{1i}>0\;\text{and}\;y_{2i}=0\right)\\ D_{i},\left(\text{if}\;y_{1i}>0\;\text{and}\;y_{2i}>0\right) \end{array}\right.$$

where

$$A_{i}=\frac{e^{x_{i}^{T}\gamma_{1}}}{1+e^{x_{i}^{T}\gamma_{1}}}\frac{e^{x_{i}^{T}\gamma_{2}}}{1+e^{x_{i}^{T}\gamma_{2}}}+\frac{e^{x_{i}^{T}\gamma_{1}}}{1+e^{x_{i}^{T}\gamma_{1}}}\frac{1}{1+e^{x_{i}^{T}\gamma_{2}}}\exp\left(\frac{-e^{x_{i}^{T}\beta_{2}}}{1+\varphi_{2}e^{x_{i}^{T}\beta_{2}}}\right)\;+M_{1}+M_{2}$$

$$M_{1}=\frac{e^{x_{i}^{T}\gamma_{2}}}{1+e^{x_{i}^{T}\gamma_{2}}}\frac{1}{1+e^{x_{i}^{T}\gamma_{1}}}\exp\left(\frac{-e^{x_{i}^{T}\beta_{1}}}{1+\varphi_{1}e^{x_{i}^{T}\beta_{1}}}\right)$$

$$M_{2}=\frac{1}{1+e^{x_{i}^{T}\gamma_{1}}}\frac{1}{1+e^{x_{i}^{T}\gamma_{2}}}\exp\left(\frac{-e^{x_{i}^{T}\beta_{1}}}{1+\varphi_{1}e^{x_{i}^{T}\beta_{1}}}-\frac{e^{x_{i}^{T}\beta_{2}}}{1+\varphi_{2}e^{x_{i}^{T}\beta_{2}}}\right)\;\times \left(1+\eta \left(1-g_{1}\right)\left(1-g_{2}\right)\right)$$

$$B_{i}=\frac{1}{1+e^{x_{i}^{T}\gamma_{2}}}{\left(\frac{e^{x_{i}^{T}\beta_{2}}}{1+\varphi_{2}e^{x_{i}^{T}\beta_{2}}}\right)}^{y_{2}}\frac{{\left(1+\varphi_{2}y_{2}\right)}^{y_{2}-1}}{y_{2}!}\;\;\times \exp\left(\frac{-e^{x_{i}^{T}\beta_{2}}\left(1+\varphi_{2}y_{2}\right)}{1+\varphi_{2}e^{x_{i}^{T}\beta_{2}}}\right)M_{3}$$

$$M_{3}=(\frac{e^{x_{i}^{T}\gamma_{1}}}{1+e^{x_{i}^{T}\gamma_{1}}}+\frac{1}{1+e^{x_{i}^{T}\gamma_{1}}}\exp\left(\frac{-e^{x_{i}^{T}\beta_{1}}}{1+\varphi_{1}e^{x_{i}^{T}\beta_{1}}}\right)\;\times \left(1+\eta \left(1-g_{1}\right)\left(e^{-y_{2}}-g_{2}\right)\right))$$

$$C_{i}=\frac{1}{1+e^{x_{i}^{T}\gamma_{1}}}{\left(\frac{e^{x_{i}^{T}\beta_{1}}}{1+\varphi_{1}e^{x_{i}^{T}\beta_{1}}}\right)}^{y_{1}}\frac{{\left(1+\varphi_{1}y_{1}\right)}^{y_{1}-1}}{y_{1}!}\;\;\times \exp\left(\frac{-e^{x_{i}^{T}\beta_{1}}\left(1+\varphi_{1}y_{1}\right)}{1+\varphi_{1}e^{x_{i}^{T}\beta_{1}}}\right)M_{4}$$

$$M_{4}=(\frac{e^{x_{i}^{T}\gamma_{2}}}{1+e^{x_{i}^{T}\gamma_{2}}}+\frac{1}{1+e^{x_{i}^{T}\gamma_{2}}}\exp\left(\frac{-e^{x_{i}^{T}\beta_{2}}}{1+\varphi_{2}e^{x_{i}^{T}\beta_{2}}}\right)\;\times \left(1+\eta \left(e^{-y_{1}}-g_{1}\right)\left(1-g_{2}\right)\right))$$

$$D_{i}=\frac{1}{1+e^{x_{i}^{T}\gamma_{1}}}\frac{1}{1+e^{x_{i}^{T}\gamma_{2}}}\prod_{l=1}^{2}\left({\left(\frac{e^{x_{i}^{T}\beta_{l}}}{1+\varphi_{l}e^{x_{i}^{T}\beta_{l}}}\right)}^{y_{l}}\frac{{\left(1+\varphi_{l}y_{l}\right)}^{y_{l}-1}}{y_{l}!}M_{5}\right)$$

$$M_{5}=\exp\left(\frac{-e^{x_{i}^{T}\beta_{l}}\left(1+\varphi_{l}y_{l}\right)}{1+\varphi_{l}e^{x_{i}^{T}\beta_{l}}}\right)\left(1+\eta \prod_{l=1}^{2}\left(e^{-y_{l}}-g_{l}\right)\right)$$

$$g_{l}=E\left(e^{-Y_{l}}\right)=\exp\left(\frac{e^{x_{i}^{T}\beta_{l}}\left(s_{l}-1\right)}{1+\varphi_{l}e^{x_{i}^{T}\beta_{l}}}\right)\;\text{with }\ln s_{l}=\frac{\varphi_{l}e^{x_{i}^{T}\beta_{l}}\left(s_{l}-1\right)}{1+\varphi_{l}e^{x_{i}^{T}\beta_{l}}}-1$$

(2) η=the multiplicative factor parameter

Parameter estimation of BZIGPR using MLE with numerical analysis of BHHH and determination of test statistics using MLRT [12].

### 2.4 GWBZIGPR

The probability density function of the GWBZIGPR is:

If $y_{1}=0\;\text{and}\;y_{2}=0$, thus $P\left(Y_{1}=0,Y_{2}=0\right)$

$$=\frac{e^{x_{i}^{T}\gamma_{1}\left(u_{i},v_{i}\right)}}{1+e^{x_{i}^{T}\gamma_{1}\left(u_{i},v_{i}\right)}}\frac{e^{x_{i}^{T}\gamma_{2}\left(u_{i},v_{i}\right)}}{1+e^{x_{i}^{T}\gamma_{2}\left(u_{i},v_{i}\right)}}+\frac{e^{x_{i}^{T}\gamma_{1}\left(u_{i},v_{i}\right)}}{1+e^{x_{i}^{T}\gamma_{1}\left(u_{i},v_{i}\right)}}\frac{1}{1+e^{x_{i}^{T}\gamma_{2}\left(u_{i},v_{i}\right)}}\exp\left(\frac{-e^{x_{i}^{T}\beta_{2}\left(u_{i},v_{i}\right)}}{1+\varphi_{2}e^{x_{i}^{T}\beta_{2}\left(u_{i},v_{i}\right)}}\right)+M_{11}+M_{12}$$

where

$$M_{11}=\frac{e^{x_{i}^{T}\gamma_{2}\left(u_{i},v_{i}\right)}}{1+e^{x_{i}^{T}\gamma_{2}\left(u_{i},v_{i}\right)}}\frac{1}{1+e^{x_{i}^{T}\gamma_{1}\left(u_{i},v_{i}\right)}}\exp\left(\frac{-e^{x_{i}^{T}\beta_{1}\left(u_{i},v_{i}\right)}}{1+\varphi_{1}e^{x_{i}^{T}\beta_{1}\left(u_{i},v_{i}\right)}}\right)$$

$$M_{12}=\frac{1}{1+e^{x_{i}^{T}\gamma_{1}\left(u_{i},v_{i}\right)}}\frac{1}{1+e^{x_{i}^{T}\gamma_{2}\left(u_{i},v_{i}\right)}}\;\exp\left(\frac{-e^{x_{i}^{T}\beta_{1}\left(u_{i},v_{i}\right)}}{1+\varphi_{1}e^{x_{i}^{T}\beta_{1}\left(u_{i},v_{i}\right)}}-\frac{e^{x_{i}^{T}\beta_{2}\left(u_{i},v_{i}\right)}}{1+\varphi_{2}e^{x_{i}^{T}\beta_{2}\left(u_{i},v_{i}\right)}}\right)\left(1+\eta \left(1-g_{1}\right)\left(1-g_{2}\right)\right)$$

If $y_{1}=0\;\text{and}\;y_{2}>0$, thus $P\left(Y_{1}=0,Y_{2}=y_{2}\right)$

$$=\frac{1}{1+e^{x_{i}^{T}\gamma_{2}\left(u_{i},v_{i}\right)}}{\left(\frac{e^{x_{i}^{T}\beta_{2}\left(u_{i},v_{i}\right)}}{1+\varphi_{2}e^{x_{i}^{T}\beta_{2}\left(u_{i},v_{i}\right)}}\right)}^{y_{2}}\frac{{\left(1+\varphi_{2}y_{2}\right)}^{y_{2}-1}}{y_{2}!}\;\;\exp\left(\frac{-e^{x_{i}^{T}\beta_{2}\left(u_{i},v_{i}\right)}\left(1+\varphi_{2}y_{2}\right)}{1+\varphi_{2}e^{x_{i}^{T}\beta_{2}\left(u_{i},v_{i}\right)}}\right)⁎M_{2}$$

where

$$M_{2}=\left(\begin{array}{c} \frac{e^{x_{i}^{T}\gamma_{1}\left(u_{i},v_{i}\right)}}{1+e^{x_{i}^{T}\gamma_{1}\left(u_{i},v_{i}\right)}}+\frac{1}{1+e^{x_{i}^{T}\gamma_{1}\left(u_{i},v_{i}\right)}}\exp\left(\frac{-e^{x_{i}^{T}\beta_{1}\left(u_{i},v_{i}\right)}}{1+\varphi_{1}e^{x_{i}^{T}\beta_{1}\left(u_{i},v_{i}\right)}}\right)\\ \left(1+\eta \left(1-g_{1}\right)\left(e^{-y_{2}}-g_{2}\right)\right) \end{array}\right)$$

If $y_{1}>0\;\text{and}\;y_{2}=0$, thus $P\left(Y_{1}=y_{1},Y_{2}=0\right)$

$$=\frac{1}{1+e^{x_{i}^{T}\gamma_{1}\left(u_{i},v_{i}\right)}}{\left(\frac{e^{x_{i}^{T}\beta_{1}\left(u_{i},v_{i}\right)}}{1+\varphi_{1}e^{x_{i}^{T}\beta_{1}\left(u_{i},v_{i}\right)}}\right)}^{y_{1}}\;\;\frac{{\left(1+\varphi_{1}y_{1}\right)}^{y_{1}-1}}{y_{1}!}\exp\left(\frac{-e^{x_{i}^{T}\beta_{1}\left(u_{i},v_{i}\right)}\left(1+\varphi_{1}y_{1}\right)}{1+\varphi_{1}e^{x_{i}^{T}\beta_{1}\left(u_{i},v_{i}\right)}}\right)⁎M_{3}$$

where

$$M_{3}=\left(\begin{array}{c} \frac{e^{x_{i}^{T}\gamma_{2}\left(u_{i},v_{i}\right)}}{1+e^{x_{i}^{T}\gamma_{2}\left(u_{i},v_{i}\right)}}+\frac{1}{1+e^{x_{i}^{T}\gamma_{2}\left(u_{i},v_{i}\right)}}\\ \exp\left(\frac{-e^{x_{i}^{T}\beta_{2}\left(u_{i},v_{i}\right)}}{1+\varphi_{2}e^{x_{i}^{T}\beta_{2}\left(u_{i},v_{i}\right)}}\right)\left(1+\eta \left(e^{-y_{1}}-g_{1}\right)\left(1-g_{2}\right)\right) \end{array}\right)$$

If $y_{1}>0\;\text{and}\;y_{2}>0$, thus $P\left(Y_{1}=y_{1},Y_{2}=y_{2}\right)$

(3) $$=\frac{1}{1+e^{x_{i}^{T}\gamma_{1}\left(u_{i},v_{i}\right)}}\frac{1}{1+e^{x_{i}^{T}\gamma_{2}\left(u_{i},v_{i}\right)}}\;\;\prod_{l=1}^{2}\left(\begin{array}{c} {\left(\frac{e^{x_{i}^{T}\beta_{l}\left(u_{i},v_{i}\right)}}{1+\varphi_{l}e^{x_{i}^{T}\beta_{l}\left(u_{i},v_{i}\right)}}\right)}^{y_{l}}\frac{{\left(1+\varphi_{l}y_{l}\right)}^{y_{l}-1}}{y_{l}!}\\ \exp\left(\frac{-e^{x_{i}^{T}\beta_{l}\left(u_{i},v_{i}\right)}\left(1+\varphi_{l}y_{l}\right)}{1+\varphi_{l}e^{x_{i}^{T}\beta_{l}\left(u_{i},v_{i}\right)}}\right)\left(1+\eta \prod_{l=1}^{2}\left(e^{-y_{l}}-g_{l}\right)\right) \end{array}\right)$$

Parameter estimation and hypothesis testing will be discussed in the result and discussion section.

*Note*: $\left(u_{i},v_{i}\right)$ denotes the latitude and longitude coordinates of the observation location.

### 2.5 Under/overdispersion detection

In statistics, underdispersion means that there was less variation in the data than predicted. Conversely, when the observed variance is higher than the variance of a theoretical model, overdispersion has occurred. Under/overdispersion detection can be done using Variance Test (VT) as follows [13]:

(4) $$\mathrm{VT}=\sum_{i=1}^{n}\frac{{\left(y_{i}-\bar{y}\right)}^{2}}{\bar{y}}$$

where $\bar{y}$ is the mean of *y*. Data are underdispersed when VT < 1, overdispersed when VT > 1, and equidispersed when VT=1.

### 2.6 Correlation and multicollinearity

Correlation testing for the response variable is carried out with the following hypothesis [14]

$H_{0}$: There is no correlation between Y_1_ and Y_2_
$H_{1}$: There is a correlation between Y_1_ and Y_2_

The test statistics is

(5) $$t=\frac{r_{y_{1},y_{2}}\sqrt{n-2}}{\sqrt{1-{\left(r_{y_{1},y_{2}}\right)}^{2}}}$$

where $r_{y_{1},y_{2}}$ is the correlation between $y_{1}$ and $y_{2}$. Reject H_0_ if $\left|t_{\mathrm{hit}}\right|>t_{\left(\alpha /2;(n-2)\right)}$. According to [15] multicollinearity can be identified by the Variance-Inflation Factor (VIF) value which is more than 10.

(6) $$\mathrm{VIF}=\frac{1}{1-R_{r}^{2}}$$

$R_{r}^{2}$ is the coefficient of determination between $x_{r}$ and the other predictor variables.

### 2.7 Spatial heterogeneity

Hypothesis testing can be done by using Glejser method, which is conducting a simultaneous test of the regression model ${\overset{⌢}{\varepsilon }}_{lr}^{2}=\beta_{10}+\beta_{11}x_{1i}+\beta_{12}x_{1i}+...+\beta_{lr}x_{ri}$. The hypothesis of Glejser test is

H_0_: $\beta_{j1}=\beta_{j2}=\ldots =\beta_{jq}=0$; j=1,2
H_1_: At least one of $\beta_{jr}\neq 0$.

The test statistics is

(7) $$G=-\left[n-q-1-\frac{1}{2}\left(j-q+1\right)\right]\ln\left(\frac{\left|{\overset{⌢}{\Sigma }}_{\Omega }\right|}{\left|{\overset{⌢}{\Sigma }}_{\omega }\right|}\right)$$

$\Sigma_{\omega }$ is a variance-covariance matrix under H_0_ and $\Sigma_{\Omega }$ is a variance-covariance matrix under population [16]. H_0_ is rejected when $G>\chi_{\left(\alpha ;lr\right)}^{2}$.

### 2.8 Weighting matrix

Weights have an important role in spatial data because the value of weight is representative of the location where each data was taken. The weight used in this study is the kernel weighting function which has a minimum GCV value.

$$\text{Fix Gaussian}\;\text{Kernel:}\;w_{ii^{⁎}}=\exp\left(-\frac{1}{2}{\left(\frac{d_{ii^{⁎}}}{h}\right)}^{2}\right)$$

$$\text{Fix bisquare kernel:}\;w_{ii^{⁎}}=\left\{ \begin{array}{cc} {\left(1-{\left(\frac{d_{ii^{⁎}}}{h_{i}}\right)}^{2}\right)}^{2},\; & d_{ii^{⁎}}\leq h_{i}\\ 0,\; & d_{ii^{⁎}}>h_{i} \end{array}\right.$$

$$\text{Adaptive Gaussian Kernel:}\;w_{ii^{⁎}}=\exp\left(-\frac{1}{2}{\left(\frac{d_{ii^{⁎}}}{h_{i}}\right)}^{2}\right)$$

(8) $$\text{Adaptive Bisquare Kernel:}\;w_{ii^{⁎}}=\left\{ \begin{array}{cc} {\left(1-{\left(\frac{d_{ii^{⁎}}}{h_{i}}\right)}^{2}\right)}^{2},\; & d_{ii^{⁎}}\leq h_{i}\\ 0,\; & d_{ii^{⁎}}>h_{i} \end{array}\right.$$

where $d_{ii^{⁎}}=\sqrt{{\left(u_{i}-u_{i^{⁎}}\right)}^{2}+{\left(v_{i}-v_{i^{⁎}}\right)}^{2}}$ is the Euclidean distance between location *i* and location $i^{⁎}$. $h_{i}$ is a smoothing parameter or bandwidth from location *i*. Selection of the optimum bandwidth can be done by Generalized Cross-Validation (GCV) method [8].

(9) $$\mathrm{GCV}=\min\left(n\sum_{i=1}^{n}\frac{{\left(y_{i}-\overset{ˆ}{y}\left(h\right)\right)}^{T}\left(y_{i}-\overset{ˆ}{y}\left(h\right)\right)}{{\left(n-v_{1}\right)}^{2}}\right)$$

$y_{i}$ is the observed value of response variable-*i*, $\overset{ˆ}{y}\left(h\right)$ is estimator value of *y*, *n* is the number of observations, $v_{1}$ is a trace (S) where $S=X{\left(X^{T}w_{i}X\right)}^{-1}X^{T}w_{i}$.

(10) $$\mathrm{AICc}=\mathrm{AIC}+\frac{2r\left(r+1\right)}{n-r-1}=-2\log\left(L\left({\overset{ˆ}{\gamma }}_{1},{\overset{ˆ}{\gamma }}_{2},{\overset{ˆ}{\beta }}_{1},{\overset{ˆ}{\beta }}_{2},{\overset{ˆ}{\varphi }}_{1},{\overset{ˆ}{\varphi }}_{2},\overset{ˆ}{\eta }\right)\right)+2r+\frac{2r\left(r+1\right)}{n-r-1}$$

*n* is the number of observations, *r* is the number of parameters estimated.

### 2.9 Maternal mortality rate

Maternal mortality according to the WHO is death during pregnancy or within period of 42 days after the end of pregnancy, which is caused by pregnancy or its handling, not caused by an accident or injury. Globally, the main causes of maternal mortality are bleeding, hypertension in pregnancy, infection, prolonged labor/obstruction, and abortus. In Indonesia, maternal mortality is dominated by bleeding, hypertension, and infection. Diseases that are indirect causes of maternal death include tuberculosis, anemia, malaria, heart disease, and others [17].

## 3 Result

### 3.1 Parameter estimation of GWBZIGPR

The first step is to construct the likelihood function as follows:

(11) $$L(\gamma_{1}\left(u_{i},v_{i}\right),\gamma_{2}\left(u_{i},v_{i}\right),\beta_{1}\left(u_{i},v_{i}\right),\beta_{2}\left(u_{i},v_{i}\right),\varphi_{1},\varphi_{2},\eta )=\prod_{i=1}^{n}{\left({\left(A_{i}\right)}^{1-b_{i}-c_{i}-d_{i}}{\left(B_{i}\right)}^{b_{i}}{\left(C_{i}\right)}^{c_{i}}{\left(D_{i}\right)}^{d_{i}}\right)}^{w_{ii^{⁎}}}$$

The second step is to construct the ln likelihood function

(12) $$l=\ln L(\gamma_{1}\left(u_{i},v_{i}\right),\gamma_{2}\left(u_{i},v_{i}\right),\beta_{1}\left(u_{i},v_{i}\right),\beta_{2}\left(u_{i},v_{i}\right),\varphi_{1},\varphi_{2},\eta )=\sum_{i=1}^{n}w_{ii^{⁎}}\left(1-b_{i}-c_{i}-d_{i}\right)\ln A_{4i}+\sum_{i=1}^{n}w_{ii^{⁎}}b_{i}\ln B_{4i}\;\;+\sum_{i=1}^{n}w_{ii^{⁎}}c_{4i}\ln C_{i}+\sum_{i=1}^{n}w_{ii^{⁎}}d_{i}\ln D_{4i}$$

The third step is constructing the first derivative of the ln-likelihood function with respect to $\gamma_{1}\left(u_{i},v_{i}\right),\gamma_{2}\left(u_{i},v_{i}\right),\beta_{1}\left(u_{i},v_{i}\right),\beta_{2}\left(u_{i},v_{i}\right),\varphi_{1},\varphi_{2},\eta$ (Appendix 2). The first derivative does not yield close-form solution so that it is completed by BHHH iteration with the step as follows:

a. Determine the initial value $\overset{ˆ}{\theta }$ and m=0 with the value ε>0 for the convergence tolerance limit
  $$\overset{ˆ}{\theta }={\left[\begin{array}{ccc} {\overset{ˆ}{\gamma }}_{1(0)}^{T}\left(u_{i},v_{i}\right) & {\overset{ˆ}{\gamma }}_{2(0)}^{T}\left(u_{i},v_{i}\right) & {\overset{ˆ}{\beta }}_{1(0)}^{T}\left(u_{i},v_{i}\right){\overset{ˆ}{\beta }}_{2(0)}^{T}\left(u_{i},v_{i}\right){\overset{ˆ}{\varphi }}_{1}{\overset{ˆ}{\varphi }}_{2}\overset{ˆ}{\eta } \end{array}\right]}^{T}$$
  where $\overset{ˆ}{\theta }$ are obtained from the univariate BZIGPR estimate.
b. Calculate the gradient vector $g\left({\overset{ˆ}{\theta }}_{m}\right)$.
c. Form a Hessian matrix: $H\left({\overset{ˆ}{\theta }}_{m}\right)=-\sum_{i=1}^{n}g_{i}\left({\overset{ˆ}{\theta }}_{m}\right)g_{i}{\left({\overset{ˆ}{\theta }}_{m}\right)}^{T}$.
d. Substitute ${\overset{ˆ}{\theta }}_{m}$ value in $g\left({\overset{ˆ}{\theta }}_{m}\right)$ and Hessian matrix.
e. Iteration starting at m=0 with the equation ${\overset{ˆ}{\theta }}_{m+1}={\overset{ˆ}{\theta }}_{m}-H^{-1}\left({\overset{ˆ}{\theta }}_{m}\right)g\left({\overset{ˆ}{\theta }}_{m}\right)$. Iteration will be stopped if $\left\|{\overset{ˆ}{\theta }}_{m+1}-{\overset{ˆ}{\theta }}_{m}\right\|\leq \varepsilon$. Repeat the b step with m=m+1.

### 3.2 Simultaneous test of GWBZIGPR

Simultaneous testing of GWBZIGPR model is carried out to determine the significance of the parameters $\beta \left(u_{i},v_{i}\right)$ and $\gamma \left(u_{i},v_{i}\right)$ simultaneously.

#### 3.2.1 Simultaneous test of $\gamma (u_{i},v_{i})$ and $\beta (u_{i},v_{i})$

The hypothesis is:

H_0_: $\beta_{j1}\left(u_{i},v_{i}\right)=...=\beta_{jq}\left(u_{i},v_{i}\right)=\gamma_{j1}\left(u_{i},v_{i}\right)=...=\gamma_{jq}\left(u_{i},v_{i}\right)=0$; j=1,2; i=1,2,...,n
H_1_: at least one of $\beta_{jr}\left(u_{i},v_{i}\right)\neq 0$

The statistics test is

(13) $$G^{2}=2\left(\log L\left(\overset{ˆ}{\Omega }\right)-\log L\left(\overset{ˆ}{\omega }\right)\right)$$

where $\ln L\left(\overset{ˆ}{\Omega }\right)$ is

(14) $$=\sum_{i=1}^{n}\left(1-b_{i}-c_{i}-d_{i}\right)\log A_{4i}^{⁎}+\sum_{i=1}^{n}b_{i}\log B_{4i}^{⁎}+\sum_{i=1}^{n}c_{i}\log C_{4i}^{⁎}\;\;+\sum_{i=1}^{n}d_{i}\log D_{4i}^{⁎}$$

The parameters in (13) are obtained from Appendix 2. $\ln L\left(\overset{ˆ}{\omega }\right)$ is a ln-likelihood function under H_0_ for the parameter $\overset{ˆ}{\omega }=\{\gamma_{10}\left(u_{i},v_{i}\right),\gamma_{20}\left(u_{i},v_{i}\right),{\overset{ˆ}{\beta }}_{10}\left(u_{i},v_{i}\right),{\overset{ˆ}{\beta }}_{20}\left(u_{i},v_{i}\right),{\overset{ˆ}{\varphi }}_{1},{\overset{ˆ}{\varphi }}_{2},\overset{ˆ}{\eta },i=1,2,...,n\}$

(15) $$\ln L\left(\overset{ˆ}{\omega }\right)=\sum_{i=1}^{n}\left(1-b_{i}-c_{i}-d_{i}\right)\log A_{5i}^{⁎}+\sum_{i=1}^{n}b_{i}\log B_{5i}^{⁎}\;\;+\sum_{i=1}^{n}c_{i}\log C_{5i}^{⁎}+\sum_{i=1}^{n}d_{i}\log D_{5i}^{⁎}$$

Reject H_0_ if $G_{\mathrm{hitung}}^{2}>\chi_{\alpha ,2nr}$. A partial test is conducted to determine which variables significantly affect the response.

#### 3.2.2 Simultaneous test of the parameter $\gamma (u_{i},v_{i})$

The hypothesis is:

H_0_: $\gamma_{j1}\left(u_{i},v_{i}\right)=...=\gamma_{jq}\left(u_{i},v_{i}\right)=0$; j=1,2; i=1,2,...,n
H_1_: at least one of $\gamma_{jq}\left(u_{i},v_{i}\right)\neq 0$

The statistics test is (13). $\mathrm{Ln}\;L\left(\overset{ˆ}{\omega }\right)$ for the parameter ***γ*** under H_0_ is:

$$\overset{ˆ}{\omega }=\left\{{\overset{ˆ}{\gamma }}_{10}\left(u_{i},v_{i}\right),{\overset{ˆ}{\gamma }}_{20}\left(u_{i},v_{i}\right),{\overset{ˆ}{\beta }}_{1}\left(u_{i},v_{i}\right),{\overset{ˆ}{\beta }}_{2}\left(u_{i},v_{i}\right),{\overset{ˆ}{\varphi }}_{1},{\overset{ˆ}{\varphi }}_{2},\overset{ˆ}{\eta },i=1,2,...,n\right\}$$

(16) $$\ln L\left(\overset{ˆ}{\omega }\right)=\sum_{i=1}^{n}\left(1-b_{i}-c_{i}-d_{i}\right)\log A_{6i}^{⁎}+\sum_{i=1}^{n}b_{i}\log B_{6i}^{⁎}\;\;+\sum_{i=1}^{n}c_{i}\log C_{6i}^{⁎}+\sum_{i=1}^{n}d_{i}\log D_{6i}^{⁎}$$

Reject H_0_ if $G_{\mathrm{hitung}}^{2}>\chi_{\alpha ,2nr}$. A partial test is conducted to determine which variables significantly affect the response.

#### 3.2.3 Simultaneous test of the parameter $\beta (u_{i},v_{i})$

The hypothesis is:

H_0_: $\beta_{j1}\left(u_{i},v_{i}\right)=...=\beta_{jq}\left(u_{i},v_{i}\right)=0$; j=1,2; i=1,2,...,n
H_1_: at least one of $\beta_{jq}\left(u_{i},v_{i}\right)\neq 0$

The statistics test is (13) where $\ln L\left(\overset{ˆ}{\omega }\right)$ is

(17) $$\ln L\left(\overset{ˆ}{\omega }\right)=\sum_{i=1}^{n}\left(1-b_{i}-c_{i}-d_{i}\right)\ln A_{7i}^{⁎}+\sum_{i=1}^{n}b_{i}\ln B_{7i}^{⁎}\;\;+\sum_{i=1}^{n}c_{i}\ln C_{7i}^{⁎}+\sum_{i=1}^{n}d_{i}\ln D_{7i}^{⁎}$$

The parameters in (17) are obtained from Appendix 3. $\mathrm{Ln}\;L\left(\overset{ˆ}{\omega }\right)$ is a ln-likelihood function under H_0_ for the parameter $\beta \left(u_{i},v_{i}\right)$ where

$$\overset{ˆ}{\omega }=\left\{{\overset{ˆ}{\gamma }}_{1}\left(u_{i},v_{i}\right),{\overset{ˆ}{\gamma }}_{2}\left(u_{i},v_{i}\right),{\overset{ˆ}{\beta }}_{10}\left(u_{i},v_{i}\right),{\overset{ˆ}{\beta }}_{20}\left(u_{i},v_{i}\right),{\overset{ˆ}{\varphi }}_{1},{\overset{ˆ}{\varphi }}_{2},\overset{ˆ}{\eta },i=1,2,...,n\right\}.$$

Reject H_0_ if $G_{\mathrm{hitung}}^{2}>\chi_{\alpha ,2nr}$.

### 3.3 The modeling of the number of pregnant maternal mortality and postpartum maternal mortality in Pekalongan Residency with GWBZIGPR

#### 3.3.1 Description of research variables

The initial step in this study was carried out by exploring the data. Based on Table 2, it shows that the number of maternal mortality both during pregnancy and postpartum in Pekalongan Residency, there are at most 3 cases. The percentage of TT2 + immunization has the greatest diversity compared to other predictor variables, namely 506.92. The percentage of obstetric complications that are treated had the smallest average of 30.17 and the percentage of K1 visits by pregnant women had the largest average of 97.89.

**Table 2:** The descriptive statistics of research variables.

Variable	Min	Max	Mean	Varian
Y_1_	0.00	3.00	0.41	0.49
Y_2_	0.00	3.00	0.67	0.91
X_1_	46.60	100.00	97.89	42.63
X_2_	49.51	100.00	92.74	50.63
X_3_	79.59	100.00	97.75	16.11
X_4_	0.64	100.00	78.22	506.92
X_5_	61.17	100.00	93.18	50.80
X_6_	10.57	61.61	30.17	92.14
X_7_	18.14	100.00	48.15	246.45

**Figure 1:**
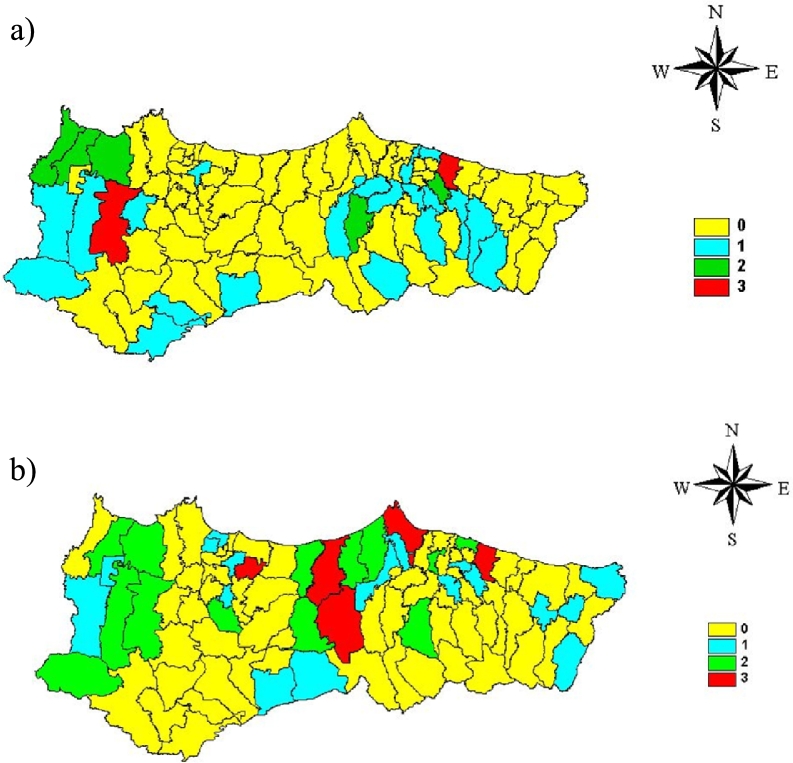
Distribution of the number of pregnant maternal mortality (a) and the number of postpartum maternal mortality (b) in Pekalongan Residency in 2017.

Sub-districts with 3 deaths of pregnant or postpartum women are marked in red, 2 deaths are marked in green, 1 death is marked in blue and zero death is marked in yellow. Based on Fig. 1, it can be seen that only 2 sub-districts have the highest number of pregnant maternal mortality, namely Batang subdistrict (Batang Regency) and Larangan subdistrict (Brebes Regency). Most of the sub-districts did not have cases of pregnant maternal mortality during 2017. Meanwhile, cases of postpartum maternal mortality spread in several sub-districts and there are 3 sub-districts in Pemalang Regency with the highest number of postpartum maternal mortality.

#### 3.3.2 Over/underdispersion detection

The results of the detection of under/overdispersion for each response have a value of VT_1_ (variance test for $y_{1}$) = 108.108 and VT_2_ (variance test for $y_{2}$) = 122.492. The value of VT_1_ and VT_2_ is more than 1 so it can be said that there is an overdispersion in the data.

#### 3.3.3 Data patterns between response variables and predictor variables

Table 3 shows that all predictor variables have a positive relationship with the number of pregnant maternal mortality. The same pattern is also shown in the variables X1, X2, X3, X4, X6 on the number of postpartum maternal mortality. However, X5 and X7 have a negative pattern, meaning that each increase in the X5 and X7 variables will reduce the number of postpartum maternal mortality in Pekalongan Residency. Further analysis using statistical tests needs to be carried out to ensure the direction of the relationship between the two responses towards the variables that influence it.

**Table 3:** Correlation coefficient and the significance of research variables.

Var	Correlation coefficient (significance)						
	X_1_	X_2_	X_3_	X_4_	X_5_	X_6_	X_7_
Y_1_	0.08	0.02	0.14	0.07	0.05	0.30	0.08
	(0.45)	(0.86)	(0.17)	(0.54)	(0.63)	(0.00)	(0.46)
Y_2_	0.21	0.11	0.16	0.11	-0.07	0.09	-0.21
	(0.05)	(0.28)	(0.14)	(0.32)	(0.52)	(0.39)	(0.04)

() = p-value.

#### 3.3.4 Response correlation test

The hypothesis is:

H_0_: There is no correlation between Y_1_ and Y_2_
H_1_: There is a correlation between Y_1_ and Y_2_

The statistics test is:

$$t=\frac{r_{y_{1},y_{2}}\sqrt{n-2}}{\sqrt{1-{\left(r_{y_{1},y_{2}}\right)}^{2}}}=\frac{0.2696\sqrt{91-2}}{\sqrt{1-0.2696^{2}}}=2.6414$$

where $r_{y_{1},y_{2}}$ is the correlation between $y_{1}$ and $y_{2}$. The t value is 2.6414 greater than $t_{\left(0.025;90\right)}=1.98$ so that the decision is to reject H_0_, or there is a relationship between the variables Y_1_ and Y_2_ so that the analysis can be continued to the next analysis, namely BZIGP.

#### 3.3.5 Multicollinearity test

A VIF value greater than 10 is evidence that there is multicollinearity between the predictor variables used [15]. The result of multicollinearity test in Table 4 concluded that there is no multicollinearity between predictor variables.

**Table 4:** The result of multicollinearity test.

	X_1_	X_2_	X_3_	X_4_	X_5_	X_6_	X_7_
VIF	2.78	2.77	1.78	1.12	1.57	1.33	1.32

The result of multicollinearity test in Table 4 concluded that there is no multicollinearity between predictor variables.

#### 3.3.6 Spatial heterogeneity test

The hypothesis is:

H_0_: $\Sigma_{1}=\Sigma_{2}=\ldots =\Sigma_{n}=\Sigma$
H_1_: at least one of $\Sigma_{i}\neq \Sigma$; i=1,2,…,91

The statistical value of the Glejser test is 90.3217, which is greater than $\chi_{\left(0,05;14\right)}^{2}=23.685$ so that the decision is to reject H_0_. This means that the number of pregnant maternal mortality and postpartum maternal mortality in Pekalongan Residency in 2017 has spatial heterogeneity between regions so that modeling with GWBZIGPR can be carried out.

#### 3.3.7 Determination of the weighting of the GWBZIGPR model

This study uses the Kernel Gaussian and Bisquare weighting functions, both fixed and adaptive. The calculation formula refers to (7). It aims to determine which bandwidth is more suitable for spatial weighting. Selection of the optimum bandwidth using the GCV method.

GWBZIGPR modeling with a fixed bisquare kernel weighting function in Table 5 yields the smallest AICc value (866.1279), so it can be concluded that the use of a fixed bisquare kernel weighting function provides a more representative weight in describing spatial heterogeneity.

**Table 5:** AICc comparison of the GWBZIGPR model based on weighting functions.

Kernel weighting functions	AICc
*Fixed Gaussian*	871.2769
*Adaptive Gaussian*	872.0369
*Fixed Bisquare*	866.1279
*Adaptive Bisquare*	917.0569

#### 3.3.8 GWBZIGPR modeling

This test aims to determine the significance of geographical factors to the model. The hypothesis used is as follows:

$H_{0}$: $\beta_{lq}\left(u_{i},v_{i}\right)=\beta_{lq},l=1,2$; q=1,2,..,7; i=1,2,...,91
$H_{1}$: at least one of $\beta_{lr}\left(u_{i},v_{i}\right)\neq \beta_{lr}$

Table 6 shows that at the significance level of α=5% the value F=89.304 is greater than $F_{0.05;14;1274}=1.669$ so that the decision is to reject H_0_ which means that there is a significant difference between the parameters of the BZIGPR model and the GWBZIGPR model. Furthermore, the similarity of the BZIGPR and GWBZIGPR models will be tested on ***γ*** parameters with the following hypothesis:

$H_{0}$: $\gamma_{lq}\left(u_{i},v_{i}\right)=\gamma_{lq},l=1,2$; q=1,2,..,7; i=1,2,...,91
$H_{1}$: at least one of $\gamma_{lq}\left(u_{i},v_{i}\right)\neq \gamma_{lq}$

Table 7 shows that at the significance level of α=5% the value of F=77.158 is greater than $F_{0.05;14;1274}=1.669$ so that the decision is to reject H_0_ which means that there is a significant difference between the parameters of the BZIGPR model and the GWBZIGPR model.

**Table 6:** Similarity test for the Poisson state model of BZIGP and GWBZIGP.

Model	Devians	df	*F*	Ftable
BZIGPR	3576.365	14	89.304	1.669
GWBZIGPR	3644.285	1274

**Table 7:** Similarity test for the zero state model of BZIGP and GWBZIGP.

Model	Devians	df	*F*	Ftabel
BZIGPR	3576.365	14	77.158	1.669
GWBZIGPR	4217.957	1274		

Furthermore, a simultaneous test was conducted to determine whether at least one predictor variable affected the number of pregnant maternal mortality and postpartum maternal mortality in the Pekalongan Residency in 2017.

Based on Table 8, the deviance value of the GWBZIGPR fixed bisquare model is greater than $\chi_{0,05;1274}^{2}=1358.15$. The conclusion is to reject H_0_ which means that at least one predictor variable has a significant effect on the model. To find out which variables affect the model, a partial test was conducted in each subdistrict. Based on partial test, for example, the parameter testing will be presented at the research location in Wanasari Subdistrict, Brebes Regency.

**Table 8:** The result of simultaneous test of GWBZIGPR model.

Par	G^2^	$\chi_{\mathrm{table}}^{2}$	Decision
***γ*** and ***β***	4265.545	1358.15	Tolak H_0_
***β***	3644.285	1358.15	Tolak H_0_
***γ***	4217.957	1358.15	Tolak H_0_

#### 3.3.9 Model selection

Based on Table 9, it can be concluded that the fix bisquare GWBZIGPR model is better than the BZIGPR model because it has a smaller AICc value.

**Table 9:** AICc comparison of the BZIGPR and GWBZIGPR model.

Model	AICc
BZIGPR	1098.138
GWBZIGPR *Fix Bisquare*	866.1279

Grouping significant variables can be seen in Appendix 4-5.

## 4 Discussion

Modeling with BZIGPR generates the same parameters for each location of observation [12]. This is different from modeling using GWBZIGPR which generates a different parameter estimator for each location. The differences in the characteristics among the location of observation, known as spatial heterogeneity, caused the factors that influence the number of deaths of pregnant women and post-partum mothers in each location to be different. Therefore, the GWBZIGPR model is more appropriate than the BZIGPR model. Although the GWBZIGPR model is more appropriate than the BZIGPR model in the case of spatial heterogeneity, in terms of computation, the GWZIGPR model is more complex than the BZIGPR model.

Based on Table 10, interpretation of the GWBZIGPR model in Wanasari Subdistrict, Brebes Regency in 2017 as follows:

a. Poisson state regression model for ${\overset{ˆ}{\mu }}_{1}$
  $$\log{\overset{ˆ}{\mu }}_{1i}=-10.4858+0.0254x_{1i}-0.0488x_{2i}+0.0212x_{3i}+m$$
  where $m=+0.0456x_{4i}-0.0026x_{5i}+0.0372x_{6i}+0.0001x_{7i}$.
  Significant variables are X_1_, X_2_, X_3_, X_4_ and X_6_ so that the interpretation of the model is as follows:
  1. Every 1 percent increase in K1 visits will increase the average number of pregnant maternal mortality by $e^{0.0254}=1.026$ times assuming the other variables are constant.
  2. Every 1 percent increase in K4 visits will reduce the average of pregnant maternal mortality by $e^{-0.0488}=0.952$ times assuming the other variables are constant.
  3. Every 1 percent increase in TT2 + immunization in pregnant women will increase the average of pregnant maternal mortality by $e^{0.0456}=1.047$ times assuming the other variables are constant.
  4. Every 1 percent increase in the handling of obstetric complications will increase the average of pregnant maternal mortality by $e^{0.0372}=1.038$ times assuming the other variables are constant.
b. Poisson state regression model for ${\overset{ˆ}{\mu }}_{2}$
  $$\log{\overset{ˆ}{\mu }}_{2i}=-30.5024+0.3468x_{1i}+0.0181x_{2i}-0.0428x_{3i}+n$$
  where $n=0.0095x_{4i}-0.0138x_{5i}+0.0124x_{6i}+0.0008x_{7i}$.
  Significant variables are X_1_, X_2_, X and X_5_ so that the interpretation of the model is as follows:
  1. Every 1 percent increase in K1 visits will increase the average number of postpartum maternal mortality by $e^{0.3468}=1.414$ times assuming the other variables are constant.
  2. Every 1 percent increase in K4 visits will increase the average number of postpartum maternal mortality by $e^{0.0181}=1.018$ times assuming the other variables are constant.
  3. Every 1 percent increase in childbirth assisted by health workers will decrease the average number of postpartum maternal mortality by $e^{-0.0428}=0.958$ times assuming the other variables are constant.
  4. Every 1 percent increase in pregnant women who received Fe3 tablet will decrease the average number of postpartum maternal mortality by $e^{-0.0138}=0.986$ times assuming the other variables are constant.
c. Zero state regression model for ${\overset{ˆ}{p}}_{1}$
  $$\mathrm{logit}\;{\overset{ˆ}{p}}_{1i}=0.01+0.0215x_{1i}+0.0167x_{2i}+0.0212x_{3i}+o_{1}$$
  where $o_{1}=0.0284x_{4i}+0.0090x_{5i}+0.0048x_{6i}-0.0138x_{7i}$.
  Significant variables are X_1_, X_2_, X_4_ and X_5_ so that the interpretation of the model is as follows:
  1. Every 1 percent increase of K1 visits will increase the chance of a zero response (no pregnant maternal mortality) by $e^{0.0215}=1.022$ times.
  2. Every 1 percent increase of K4 visit will increase the chance of a zero response (no pregnant maternal mortality) by $e^{0.0167}=1.017$ times.
  3. Every 1 percent increase of pregnant women who get TT2 + immunization will increase the chance of a zero response (no pregnant maternal mortality) by $e^{0,0284}=1,029$ times.
  4. Every 1 percent increase of pregnant women who get Fe3 tablet will increase the chance of a zero response (no pregnant maternal mortality) by $e^{0,0090}=1,009$ times.
d. Zero state regression model for ${\overset{ˆ}{p}}_{2}$
  $$\mathrm{logit}\;{\overset{ˆ}{p}}_{2i}=0,0120+0,0132x_{1i}+0,0102x_{2i}+0,0129x_{3i}+q_{1}$$
  where $q_{1}=-0,0017x_{4i}+0,0160x_{5i}-0,0093x_{6i}+0,0129x_{7i}$.
  Significant variables are X_1_, X_2_, X_3_, and X_5_ so that the interpretation of the model is as follows:
  1. Every 1 percent increase of K1 visits will increase the chance of a zero response (no postpartum maternal mortality) by $e^{0,0132}=1,0133$ times.
  2. Every 1 percent increase of K4 visits will increase the chance of a zero response (no postpartum maternal mortality) by $e^{0.0102}=1.0102$ times.
  3. Every 1 percent increase of childbirth assisted by health workers will increase the chance of a zero response (no postpartum maternal mortality) by $e^{0.0129}=1.0130$ times.
  4. Every 1 percent increase of pregnant women who received Fe3 tablet will increase the chance of a zero response (no postpartum maternal mortality) by $e^{0.0160}=1.0161$ times.

**Table 10:** The result of the GWBZIGPR parameter estimation in Wanasari District.

Parameter	Est. Value	SE	Z	P-*Value*
*γ*_10_	0.0100	0.0000	341.7580	0.0000
*γ*_11_	0.0215	0.0042	5.0819	0.0000
*γ*_12_	0.0167	0.0025	6.7874	0.0000
*γ*_13_	0.0212	0.0044	4.8398	0.0000
*γ*_14_	0.0284	0.0099	2.8765	0.0040
*γ*_15_	0.0090	0.0035	2.5425	0.0110
*γ*_16_	0.0048	0.0106	0.4470	0.6549
*γ*_17_	-0.0138	0.0088	-1.5722	0.1159
*γ*_20_	0.0120	0.0000	268.2501	0.0000
*γ*_21_	0.0132	0.0046	2.8762	0.0040
*γ*_22_	0.0102	0.0037	2.7582	0.0058
*γ*_23_	0.0129	0.0039	3.3253	0.0009
*γ*_24_	0.0017	0.0083	0.2042	0.8382
*γ*_25_	0.0160	0.0060	2.6469	0.0081
*γ*_26_	-0.0093	0.0153	-0.6090	0.5425
*γ*_27_	0.0129	0.0102	1.2578	0.2085
*β*_10_	-10.4858	0.0001	-92104.9306	0.0000
*β*_11_	0.0254	0.0025	10.3476	0.0000
*β*_12_	-0.0488	0.0024	-20.6242	0.0000
*β*_13_	0.0644	0.0022	29.7909	0.0000
*β*_14_	0.0456	0.0153	2.9766	0.0029
*β*_15_	-0.0026	0.0047	-0.5635	0.5731
*β*_16_	0.0372	0.0057	6.5463	0.0000
*β*_17_	0.0001	0.0070	0.0092	0.9926
*β*_20_	-30.5024	0.0001	-365651.0969	0.0000
*β*_21_	0.3468	0.0044	79.5381	0.0000
*β*_22_	0.0181	0.0041	4.4351	0.0000
*β*_23_	-0.0428	0.0056	-7.6620	0.0000
*β*_24_	0.0095	0.0149	0.6340	0.5261
*β*_25_	-0.0138	0.0065	-2.1359	0.0327
*β*_26_	0.0124	0.0086	1.4498	0.1471
*β*_27_	0.0008	0.0137	0.0558	0.9555

Significance level α=5%.

Most of the sign parameter coefficients do not fit the theory. For example, the increase in the percentage of the predictor variable is expected to reduce the number of maternal deaths but in fact increase the number of maternal deaths. This is thought to be due to the data used in this study is cross-section data so that the health program carried out by the government in 2017 did not have a direct impact on that year but had an impact on the following year. Based on a deep interview with the Central Java Provincial Health Office, a pregnant woman is counted as having a K1 visit or a K4 visit when the pregnant woman visits to have her pregnancy checked at a health facility. In K1 and K4 visits, 10 examinations must be carried out by health workers, starting from measuring height to management, getting treatment so that when the pregnant woman has only carried out 1 examination or 10 examinations, it is still counted as 1 visit. By knowing the history of examinations, the cause of maternal death can be anticipated, but there are no benchmarks to calculate the quality of pregnancy visits based on these 10 examinations.

Previous studies in the same area but different regions and methods have been carried out by Aeni (2013). The results of the study showed that maternal death was influenced by obstetric complications and antenatal care. Pregnant women with obstetric complications have a risk of death 12,198 times greater than pregnant women without obstetric complications, and incomplete antenatal care will increase the risk of maternal death up to 7.86 times [18].

## 5 Conclusions

The following are the conclusions obtained based on the analysis of the GWBZIGPR models.

1. Parameter estimation using MLE does not yield a closed-form solution so that numerical iteration is carried out using the BHHH method.
2. The fix bisquare kernel weighting function yields the smallest AICc value among other kernel weighting functions so it is used for the GWBZIGPR model.
3. The AICc value of the GWBZIGPR model is smaller than the BZIGPR model so that the GWBZIGPR model is better for modeling the number of pregnant maternal mortality and postpartum maternal mortality in Pekalongan Residency.
4. Modeling using GWBZIGPR in the zero state model resulted in 6 sub-district groups based on the significant similarity of variables to the number of pregnant maternal mortality and 8 sub-district groups based on the similarity of variables that were significant to the number of postpartum maternal mortality. Meanwhile, modeling using GWBZIGPR in the Poisson state model resulted in 6 sub-district groups based on the significant similarity of variables to the number of pregnant maternal mortality and 6 sub-district groups based on the significant similarity of variables to the number of postpartum maternal mortality.
5. To anticipate the causes of maternal mortality, the local government should pay more attention to every procedure in antenatal care to handling childbirth because so far there is no measure to determine the quality of antenatal care. Each procedure should be recorded which later can be used as a benchmark in determining whether a pregnant/postpartum mother is categorized as getting complete health service.

## Declarations

### Author contribution statement

Purhadi, Irhamah: Conceived and designed the experiments.

D. N. Sari, Q. Aini: Analyzed and interpreted the data; Wrote the paper.

### Funding statement

This research is funded by the Deputy for Research and Development Strengthening, The Ministry of Research and Technology/The National Research and Innovation Agency with grant number: 3/E1/KP.PTNBH/2020.

### Data availability statement

The authors do not have permission to share data.

### Declaration of interests statement

The authors declare no conflict of interest.

### Additional information

Supplementary content related to this article has been published online at https://doi.org/10.1016/j.heliyon.2021.e07491.

No additional information is available for this paper.

## Supplementary material

The following Supplementary material is associated with this article:

Clean copy of GWBZIGPR (revision2)-appendices.pdf Appendices. Clean copy of GWBZIGPR (revision2)-appendices.pdf
