# Supplementary material for: Geographically weighted bivariate zero inflated generalized Poisson regression model and its application
Source: Heliyon. 2021 Jul 8;7(7):e07491. doi: 10.1016/j.heliyon.2021.e07491 (PMC8319482; doi:10.1016/j.heliyon.2021.e07491)
Supplement: Clean copy of GWBZIGPR (revision2)-appendices.pdf — Appendices. [file mmc1.pdf]

Appendix 1: First Derivative of Log Likelihood Function (GWBZIGPR)

$$\frac{\partial l}{\partial \boldsymbol{\gamma}_1\left(u_{i^*},v_{i^*}\right)}=\sum_{i=1}^{n} w_{ii^*}\left(1-b_i-c_i-d_i\right)\left(-\frac{\mathrm{e}^{\mathbf{x}_i^T\boldsymbol{\gamma}_1\left(u_{i^*},v_{i^*}\right)}\mathbf{x}_i^T}{\left(1+e^{\mathbf{x}_i^T\boldsymbol{\gamma}_1\left(u_{i^*},v_{i^*}\right)}\right)\left(1+e^{\mathbf{x}_i^T\boldsymbol{\gamma}_2\left(u_{i^*},v_{i^*}\right)}\right)}+N\right)$$

$$+\sum_{i=1}^{n} w_{ii^*}b_i\left(-\frac{e^{\mathbf{x}_i^T\boldsymbol{\gamma}_1\left(u_{i^*},v_{i^*}\right)}\mathbf{x}_i^T}{\left(1+e^{\mathbf{x}_i^T\boldsymbol{\gamma}_1\left(u_{i^*},v_{i^*}\right)}\right)}+\frac{e^{\mathbf{x}_i^T\boldsymbol{\gamma}_1\left(u_{i^*},v_{i^*}\right)}\mathbf{x}_i^T}{\left(e^{\mathbf{x}_i^T\boldsymbol{\gamma}_1\left(u_{i^*},v_{i^*}\right)}+\exp\left(\frac{-e^{\mathbf{x}_i^T\boldsymbol{\beta}_1\left(u_{i^*},v_{i^*}\right)}}{1+\varphi_1 e^{\mathbf{x}_i^T\boldsymbol{\beta}_1\left(u_{i^*},v_{i^*}\right)}}\right)\left(1+\eta\left(1-g_1\right)\left(e^{-y_{2i}}-g_2\right)\right)\right)}\right)$$

$$+\sum_{i=1}^{n} w_{ii^*}c_i\left(-\frac{e^{\mathbf{x}_i^T\boldsymbol{\gamma}_1\left(u_{i^*},v_{i^*}\right)}\mathbf{x}_i^T}{\left(1+e^{\mathbf{x}_i^T\boldsymbol{\gamma}_1\left(u_{i^*},v_{i^*}\right)}\right)}\right)+\sum_{i=1}^{n} w_{ii^*}d_i\left(-\frac{e^{\mathbf{x}_i^T\boldsymbol{\gamma}_1\left(u_{i^*},v_{i^*}\right)}\mathbf{x}_i^T}{\left(1+e^{\mathbf{x}_i^T\boldsymbol{\gamma}_1\left(u_{i^*},v_{i^*}\right)}\right)}\right)$$

where

$$N=\frac{e^{\mathbf{x}_i^T\boldsymbol{\gamma}_1\left(u_{i^*},v_{i^*}\right)}\left(e^{\mathbf{x}_i^T\boldsymbol{\gamma}_2\left(u_{i^*},v_{i^*}\right)}+\exp\left(\frac{-e^{\mathbf{x}_i^T\boldsymbol{\beta}_2\left(u_{i^*},v_{i^*}\right)}}{1+\varphi_2 e^{\mathbf{x}_i^T\boldsymbol{\beta}_2\left(u_{i^*},v_{i^*}\right)}}\right)\right)\mathbf{x}_i^T}{e^{\mathbf{x}_i^T\boldsymbol{\gamma}_1\left(u_{i^*},v_{i^*}\right)}\left(e^{\mathbf{x}_i^T\boldsymbol{\gamma}_2\left(u_{i^*},v_{i^*}\right)}+\exp\left(\frac{-e^{\mathbf{x}_i^T\boldsymbol{\beta}_2\left(u_{i^*},v_{i^*}\right)}}{1+\varphi_2 e^{\mathbf{x}_i^T\boldsymbol{\beta}_2\left(u_{i^*},v_{i^*}\right)}}\right)\right)+e^{\mathbf{x}_i^T\boldsymbol{\gamma}_2\left(u_{i^*},v_{i^*}\right)}\exp\left(\frac{-e^{\mathbf{x}_i^T\boldsymbol{\beta}_1\left(u_{i^*},v_{i^*}\right)}}{1+\varphi_1 e^{\mathbf{x}_i^T\boldsymbol{\beta}_1\left(u_{i^*},v_{i^*}\right)}}\right)+\exp\left(\frac{-e^{\mathbf{x}_i^T\boldsymbol{\beta}_1\left(u_{i^*},v_{i^*}\right)}}{1+\varphi_1 e^{\mathbf{x}_i^T\boldsymbol{\beta}_1\left(u_{i^*},v_{i^*}\right)}}-\frac{e^{\mathbf{x}_i^T\boldsymbol{\beta}_2\left(u_{i^*},v_{i^*}\right)}}{1+\varphi_2\,\mathrm{e}^{\mathbf{x}_i^T\boldsymbol{\beta}_2\left(u_{i^*},v_{i^*}\right)}}\right)N_1}$$

$$N_1=\left(1+\eta\left(1-g_1\right)\left(1-g_2\right)\right)$$

$$\frac{\partial l}{\partial \boldsymbol{\gamma}_2\left(u_{i^*},v_{i^*}\right)}=\sum_{i=1}^{n} w_{ii^*}\left(1-b_i-c_i-d_i\right)\left(-\frac{e^{\mathbf{x}_i^T\boldsymbol{\gamma}_2\left(u_{i^*},v_{i^*}\right)}\mathbf{x}_i^T}{\left(1+e^{\mathbf{x}_i^T\boldsymbol{\gamma}_2\left(u_{i^*},v_{i^*}\right)}\right)\left(1+e^{\mathbf{x}_i^T\boldsymbol{\gamma}_1\left(u_{i^*},v_{i^*}\right)}\right)}+\frac{e^{\mathbf{x}_i^T\boldsymbol{\gamma}_2\left(u_{i^*},v_{i^*}\right)}\left(e^{\mathbf{x}_i^T\boldsymbol{\gamma}_1\left(u_{i^*},v_{i^*}\right)}+\exp\left(\frac{-e^{\mathbf{x}_i^T\boldsymbol{\beta}_1\left(u_{i^*},v_{i^*}\right)}}{1+\varphi_1 e^{\mathbf{x}_i^T\boldsymbol{\beta}_1\left(u_{i^*},v_{i^*}\right)}}\right)\right)\mathbf{x}_i^T}{e^{\mathbf{x}_i^T\boldsymbol{\gamma}_1\left(u_{i^*},v_{i^*}\right)}\left(\mathrm{e}^{\mathbf{x}_i^T\boldsymbol{\gamma}_2\left(u_{i^*},v_{i^*}\right)}+\exp\left(\frac{-e^{\mathbf{x}_i^T\boldsymbol{\beta}_2\left(u_{i^*},v_{i^*}\right)}}{1+\varphi_2 e^{\mathbf{x}_i^T\boldsymbol{\beta}_2\left(u_{i^*},v_{i^*}\right)}}\right)\right)+e^{\mathbf{x}_i^T\boldsymbol{\gamma}_2\left(u_{i^*},v_{i^*}\right)}\exp\left(\frac{-e^{\mathbf{x}_i^T\boldsymbol{\beta}_1\left(u_{i^*},v_{i^*}\right)}}{1+\varphi_1 e^{\mathbf{x}_i^T\boldsymbol{\beta}_1\left(u_{i^*},v_{i^*}\right)}}\right)+N}\right)$$

$$+\sum_{i=1}^{n} w_{ii^*}b_i\left(-\frac{e^{\mathbf{x}_i^T\boldsymbol{\gamma}_2\left(u_{i^*},v_{i^*}\right)}\mathbf{x}_i^T}{\left(1+e^{\mathbf{x}_i^T\boldsymbol{\gamma}_2\left(u_{i^*},v_{i^*}\right)}\right)}\right)+\sum_{i=1}^{n} w_{ii^*}c_i\left(-\frac{e^{\mathbf{x}_i^T\boldsymbol{\gamma}_2\left(u_{i^*},v_{i^*}\right)}\mathbf{x}_i^T}{\left(1+e^{\mathbf{x}_i^T\boldsymbol{\gamma}_2\left(u_{i^*},v_{i^*}\right)}\right)}+\frac{e^{\mathbf{x}_i^T\boldsymbol{\gamma}_2\left(u_{i^*},v_{i^*}\right)}\mathbf{x}_i^T}{\left(e^{\mathbf{x}_i^T\boldsymbol{\gamma}_2\left(u_{i^*},v_{i^*}\right)}+\exp\left(\frac{-e^{\mathbf{x}_i^T\boldsymbol{\beta}_2\left(u_{i^*},v_{i^*}\right)}}{1+\varphi_2 e^{\mathbf{x}_i^T\boldsymbol{\beta}_2\left(u_{i^*},v_{i^*}\right)}}\right)\left(1+\eta\left(1-g_2\right)\left(e^{-y_{1i}}-g_1\right)\right)\right)}\right)+\sum_{i=1}^{n} w_{ii^*}d_i\left(-\frac{e^{\mathbf{x}_i^T\boldsymbol{\gamma}_2\left(u_{i^*},v_{i^*}\right)}\mathbf{x}_i^T}{\left(1+e^{\mathbf{x}_i^T\boldsymbol{\gamma}_2\left(u_{i^*},v_{i^*}\right)}\right)}\right)$$

where $N=\exp\left(\frac{-e^{\mathbf{x}_i^T\boldsymbol{\beta}_1\left(u_{i^*},v_{i^*}\right)}}{1+\varphi_1 e^{\mathbf{x}_i^T\boldsymbol{\beta}_1\left(u_{i^*},v_{i^*}\right)}}-\frac{e^{\mathbf{x}_i^T\boldsymbol{\beta}_2\left(u_{i^*},v_{i^*}\right)}}{1+\varphi_2\,\mathrm{e}^{\mathbf{x}_i^T\boldsymbol{\beta}_2\left(u_{i^*},v_{i^*}\right)}}\right)\left(1+\eta\left(1-g_1\right)\left(1-g_2\right)\right)$

$$\frac{\partial l}{\partial \boldsymbol{\beta}_1\left(u_{i^*},v_{i^*}\right)}=\sum_{i=1}^{n} w_{ii^*}\left(1-b_i-c_i-d_i\right)\left(\frac{1}{S}\left(e^{\mathbf{x}_i^T\boldsymbol{\gamma}_2\left(u_{i^*},v_{i^*}\right)}\exp\left(\frac{-e^{\mathbf{x}_i^T\boldsymbol{\beta}_1\left(u_{i^*},v_{i^*}\right)}}{1+\varphi_1 e^{\mathbf{x}_i^T\boldsymbol{\beta}_1\left(u_{i^*},v_{i^*}\right)}}\right)\left(\frac{-e^{\mathbf{x}_i^T\boldsymbol{\beta}_1\left(u_{i^*},v_{i^*}\right)}\mathbf{x}_i^T}{1+\varphi_1 e^{\mathbf{x}_i^T\boldsymbol{\beta}_1\left(u_{i^*},v_{i^*}\right)}}+\frac{\left(e^{\mathbf{x}_i^T\boldsymbol{\beta}_1\left(u_{i^*},v_{i^*}\right)}\right)^2\varphi_1\mathbf{x}_i^T}{\left(1+\varphi_1 e^{\mathbf{x}_i^T\boldsymbol{\beta}_1\left(u_{i^*},v_{i^*}\right)}\right)^2}\right)+OT\frac{\partial g_1}{\partial\boldsymbol{\beta}_1\left(u_{i^*},v_{i^*}\right)}\right)\right)$$

$$+\sum_{i=1}^{n} w_{ii^*}b_i\left(\frac{\exp\left(\frac{-e^{\mathbf{x}_i^T\boldsymbol{\beta}_1\left(u_{i^*},v_{i^*}\right)}}{1+\varphi_1 e^{\mathbf{x}_i^T\boldsymbol{\beta}_1\left(u_{i^*},v_{i^*}\right)}}\right)\left(1+\eta\left(1-g_1\right)\left(e^{-y_{2i}}-g_2\right)\right)\left(\frac{-e^{\mathbf{x}_i^T\boldsymbol{\beta}_1\left(u_{i^*},v_{i^*}\right)}\mathbf{x}_i^T}{1+\varphi_1 e^{\mathbf{x}_i^T\boldsymbol{\beta}_1\left(u_{i^*},v_{i^*}\right)}}-\frac{\left(e^{\mathbf{x}_i^T\boldsymbol{\beta}_1\left(u_{i^*},v_{i^*}\right)}\right)^2\varphi_1\mathbf{x}_i^T}{\left(1+\varphi_1 e^{\mathbf{x}_i^T\boldsymbol{\beta}_1\left(u_{i^*},v_{i^*}\right)}\right)^2}\right)}{\left(e^{\mathbf{x}_i^T\boldsymbol{\gamma}_1\left(u_{i^*},v_{i^*}\right)}+\exp\left(\frac{-e^{\mathbf{x}_i^T\boldsymbol{\beta}_1\left(u_{i^*},v_{i^*}\right)}}{1+\varphi_1 e^{\mathbf{x}_i^T\boldsymbol{\beta}_1\left(u_{i^*},v_{i^*}\right)}}\right)\left(1+\eta\left(1-g_1\right)\left(e^{-y_{2i}}-g_2\right)\right)\right)}\frac{\partial g_1}{\partial\boldsymbol{\beta}_1\left(u_{i^*},v_{i^*}\right)}\right)$$

$$+\sum_{i=1}^{n} w_{ii^*}c_i\left(y_{1i}\left(\mathbf{x}_i^T-\frac{e^{\mathbf{x}_i^T\boldsymbol{\beta}_1\left(u_{i^*},v_{i^*}\right)}\varphi_1\mathbf{x}_i^T}{\left(1+\varphi_1 e^{\mathbf{x}_i^T\boldsymbol{\beta}_1\left(u_{i^*},v_{i^*}\right)}\right)}\right)+\left(\frac{-e^{\mathbf{x}_i^T\boldsymbol{\beta}_1\left(u_{i^*},v_{i^*}\right)}\left(1+\varphi_1 y_{1i}\right)\mathbf{x}_i^T}{1+\varphi_1 e^{\mathbf{x}_i^T\boldsymbol{\beta}_1\left(u_{i^*},v_{i^*}\right)}}-\frac{\left(e^{\mathbf{x}_i^T\boldsymbol{\beta}_1\left(u_{i^*},v_{i^*}\right)}\right)^2\left(1+\varphi_1 y_{1i}\right)\varphi_1\mathbf{x}_i^T}{\left(1+\varphi_1 e^{\mathbf{x}_i^T\boldsymbol{\beta}_1\left(u_{i^*},v_{i^*}\right)}\right)^2}\right)+P\frac{\partial g_1}{\partial\boldsymbol{\beta}_1\left(u_{i^*},v_{i^*}\right)}\right)$$

$$+\sum_{i=1}^{n} w_{ii^*}d_i\left(y_{1i}\mathbf{x}_i^T-\frac{y_{1i}\varphi_1 e^{\mathbf{x}_i^T\boldsymbol{\beta}_1\left(u_{i^*},v_{i^*}\right)}\mathbf{x}_i^T}{\left(1+\varphi_1 e^{\mathbf{x}_i^T\boldsymbol{\beta}_1\left(u_{i^*},v_{i^*}\right)}\right)}+\left(\frac{-e^{\mathbf{x}_i^T\boldsymbol{\beta}_1\left(u_{i^*},v_{i^*}\right)}\left(1+\varphi_1 y_{1i}\right)\mathbf{x}_i^T}{1+\varphi_1 e^{\mathbf{x}_i^T\boldsymbol{\beta}_1\left(u_{i^*},v_{i^*}\right)}}\left(1-\frac{\varphi_1 e^{\mathbf{x}_i^T\boldsymbol{\beta}_1\left(u_{i^*},v_{i^*}\right)}}{\left(1+\varphi_1 e^{\mathbf{x}_i^T\boldsymbol{\beta}_1\left(u_{i^*},v_{i^*}\right)}\right)}\right)\right)+\frac{1}{\left(1+\eta\left(e^{-y_{1i}}-g_1\right)\left(e^{-y_{2i}}-g_2\right)\right)}\frac{\partial g_1}{\partial\boldsymbol{\beta}_1\left(u_{i^*},v_{i^*}\right)}\right)$$

where

$$S=e^{\mathbf{x}_i^T\boldsymbol{\gamma}_1\left(u_{i^*},v_{i^*}\right)}\left(e^{\mathbf{x}_i^T\boldsymbol{\gamma}_2\left(u_{i^*},v_{i^*}\right)}+\exp\left(\frac{-e^{\mathbf{x}_i^T\boldsymbol{\beta}_2\left(u_{i^*},v_{i^*}\right)}}{1+\varphi_2 e^{\mathbf{x}_i^T\boldsymbol{\beta}_2\left(u_{i^*},v_{i^*}\right)}}\right)\right)+e^{\mathbf{x}_i^T\boldsymbol{\gamma}_2\left(u_{i^*},v_{i^*}\right)}\exp\left(\frac{-e^{\mathbf{x}_i^T\boldsymbol{\beta}_1\left(u_{i^*},v_{i^*}\right)}}{1+\varphi_1 e^{\mathbf{x}_i^T\boldsymbol{\beta}_1\left(u_{i^*},v_{i^*}\right)}}\right)+\exp\left(\frac{-e^{\mathbf{x}_i^T\boldsymbol{\beta}_1\left(u_{i^*},v_{i^*}\right)}}{1+\varphi_1 e^{\mathbf{x}_i^T\boldsymbol{\beta}_1\left(u_{i^*},v_{i^*}\right)}}-\frac{e^{\mathbf{x}_i^T\boldsymbol{\beta}_2\left(u_{i^*},v_{i^*}\right)}}{1+\varphi_2\,\mathrm{e}^{\mathbf{x}_i^T\boldsymbol{\beta}_2\left(u_{i^*},v_{i^*}\right)}}\right)S_1$$

$$S_1=\left(1+\eta\left(1-g_1\right)\left(1-g_2\right)\right)$$

$$O=\exp\left(\frac{-e^{\mathbf{x}_i^T\boldsymbol{\beta}_1\left(u_{i^*},v_{i^*}\right)}}{1+\varphi_1 e^{\mathbf{x}_i^T\boldsymbol{\beta}_1\left(u_{i^*},v_{i^*}\right)}}-\frac{e^{\mathbf{x}_i^T\boldsymbol{\beta}_2\left(u_{i^*},v_{i^*}\right)}}{1+\varphi_2 e^{\mathbf{x}_i^T\boldsymbol{\beta}_2\left(u_{i^*},v_{i^*}\right)}}\right)\left(\frac{-e^{\mathbf{x}_i^T\boldsymbol{\beta}_1\left(u_{i^*},v_{i^*}\right)}\mathbf{x}_i^T}{1+\varphi_1 e^{\mathbf{x}_i^T\boldsymbol{\beta}_1\left(u_{i^*},v_{i^*}\right)}}-\frac{\left(e^{\mathbf{x}_i^T\boldsymbol{\beta}_1\left(u_{i^*},v_{i^*}\right)}\right)^2\varphi_1\mathbf{x}_i^T}{\left(1+\varphi_1 e^{\mathbf{x}_i^T\boldsymbol{\beta}_1\left(u_{i^*},v_{i^*}\right)}\right)^2}\right)$$

$$P=\frac{1}{\left(e^{\mathbf{x}_i^T\boldsymbol{\gamma}_2\left(u_{i^*},v_{i^*}\right)}+\exp\left(\frac{-e^{\mathbf{x}_i^T\boldsymbol{\beta}_2\left(u_{i^*},v_{i^*}\right)}}{1+\varphi_2 e^{\mathbf{x}_i^T\boldsymbol{\beta}_2\left(u_{i^*},v_{i^*}\right)}}\right)\left(1+\eta\left(1-g_2\right)\left(e^{-y_{1i}}-g_1\right)\right)\right)}$$

$$
\frac{\partial l}{\partial \boldsymbol{\beta}_2\left(u_{i^*},v_{i^*}\right)}=\sum_{i=1}^{n} w_{ii^*}\left(1-b_i-c_i-d_i\right)\left(\frac{1}{S}\left(e^{\mathbf{x}_i^T\boldsymbol{\gamma}_1\left(u_{i^*},v_{i^*}\right)}\exp\left(\frac{-e^{\mathbf{x}_i^T\boldsymbol{\beta}_2\left(u_{i^*},v_{i^*}\right)}}{1+\varphi_2 e^{\mathbf{x}_i^T\boldsymbol{\beta}_2\left(u_{i^*},v_{i^*}\right)}}\right)\left(\frac{-e^{\mathbf{x}_i^T\boldsymbol{\beta}_2\left(u_{i^*},v_{i^*}\right)}\mathbf{x}_i^T}{1+\varphi_2 e^{\mathbf{x}_i^T\boldsymbol{\beta}_2\left(u_{i^*},v_{i^*}\right)}}+\frac{\left(e^{\mathbf{x}_i^T\boldsymbol{\beta}_2\left(u_{i^*},v_{i^*}\right)}\right)^2\varphi_2\mathbf{x}_i^T}{\left(1+\varphi_2 e^{\mathbf{x}_i^T\boldsymbol{\beta}_2\left(u_{i^*},v_{i^*}\right)}\right)^2}\right)+U\right)\right)
$$

$$
+\sum_{i=1}^{n} w_{ii^*}b_i\left(y_{2i}\mathbf{x}_i^T-\frac{y_{2i}\varphi_2 e^{\mathbf{x}_i^T\boldsymbol{\beta}_2\left(u_{i^*},v_{i^*}\right)}\mathbf{x}_i^T}{\left(1+\varphi_2 e^{\mathbf{x}_i^T\boldsymbol{\beta}_2\left(u_{i^*},v_{i^*}\right)}\right)}+\left(\frac{-e^{\mathbf{x}_i^T\boldsymbol{\beta}_2\left(u_{i^*},v_{i^*}\right)}\left(1+\varphi_2 y_{2i}\right)\mathbf{x}_i^T}{1+\varphi_2 e^{\mathbf{x}_i^T\boldsymbol{\beta}_2\left(u_{i^*},v_{i^*}\right)}}-\frac{\left(e^{\mathbf{x}_i^T\boldsymbol{\beta}_2\left(u_{i^*},v_{i^*}\right)}\right)^2\left(1+\varphi_2 y_{2i}\right)\varphi_2\mathbf{x}_i^T}{\left(1+\varphi_2 e^{\mathbf{x}_i^T\boldsymbol{\beta}_2\left(u_{i^*},v_{i^*}\right)}\right)^2}\right)+V\frac{\partial g_2}{\partial \boldsymbol{\beta}_2\left(u_{i^*},v_{i^*}\right)}\right)
$$

$$
+\sum_{i=1}^{n} w_{ii^*}c_i\left(\frac{\exp\left(\frac{-e^{\mathbf{x}_i^T\boldsymbol{\beta}_2\left(u_{i^*},v_{i^*}\right)}}{1+\varphi_2 e^{\mathbf{x}_i^T\boldsymbol{\beta}_2\left(u_{i^*},v_{i^*}\right)}}\right)\left(1+\eta\left(1-g_2\right)\left(e^{-y_{1i}}-g_1\right)\right)\left(\frac{-e^{\mathbf{x}_i^T\boldsymbol{\beta}_2\left(u_{i^*},v_{i^*}\right)}\mathbf{x}_i^T}{1+\varphi_2 e^{\mathbf{x}_i^T\boldsymbol{\beta}_2\left(u_{i^*},v_{i^*}\right)}}-\frac{\left(e^{\mathbf{x}_i^T\boldsymbol{\beta}_2\left(u_{i^*},v_{i^*}\right)}\right)^2\varphi_2\mathbf{x}_i^T}{\left(1+\varphi_2 e^{\mathbf{x}_i^T\boldsymbol{\beta}_2\left(u_{i^*},v_{i^*}\right)}\right)^2}\right)}{\left(e^{\mathbf{x}_i^T\boldsymbol{\gamma}_2\left(u_{i^*},v_{i^*}\right)}+\exp\left(\frac{-e^{\mathbf{x}_i^T\boldsymbol{\beta}_2\left(u_{i^*},v_{i^*}\right)}}{1+\varphi_2 e^{\mathbf{x}_i^T\boldsymbol{\beta}_2\left(u_{i^*},v_{i^*}\right)}}\right)\left(1+\eta\left(1-g_2\right)\left(e^{-y_{1i}}-g_1\right)\right)\right)}\frac{\partial g_2}{\partial \boldsymbol{\beta}_2\left(u_{i^*},v_{i^*}\right)}\right)
$$

$$
+\sum_{i=1}^{n} w_{ii^*}d_i\left(y_{2i}\mathbf{x}_i^T-\frac{y_{2i}\varphi_2 e^{\mathbf{x}_i^T\boldsymbol{\beta}_2\left(u_{i^*},v_{i^*}\right)}\mathbf{x}_i^T}{\left(1+\varphi_2 e^{\mathbf{x}_i^T\boldsymbol{\beta}_2\left(u_{i^*},v_{i^*}\right)}\right)}+\left(\frac{-e^{\mathbf{x}_i^T\boldsymbol{\beta}_2\left(u_{i^*},v_{i^*}\right)}\left(1+\varphi_2 y_{2i}\right)\mathbf{x}_i^T}{1+\varphi_2 e^{\mathbf{x}_i^T\boldsymbol{\beta}_2\left(u_{i^*},v_{i^*}\right)}}\left(1-\frac{\varphi_2 e^{\mathbf{x}_i^T\boldsymbol{\beta}_2\left(u_{i^*},v_{i^*}\right)}}{\left(1+\varphi_2 e^{\mathbf{x}_i^T\boldsymbol{\beta}_2\left(u_{i^*},v_{i^*}\right)}\right)}\right)\right)+\frac{1}{\left(1+\eta\left(e^{-y_{1i}}-g_1\right)\left(e^{-y_{2i}}-g_2\right)\right)}\frac{\partial g_2}{\partial \beta_2\left(u_{i^*},v_{i^*}\right)}\right)
$$

where $V=\dfrac{1}{\left(e^{\mathbf{x}_i^T\boldsymbol{\gamma}_1\left(u_{i^*},v_{i^*}\right)}+\exp\left(\frac{-e^{\mathbf{x}_i^T\boldsymbol{\beta}_1\left(u_{i^*},v_{i^*}\right)}}{1+\varphi_1 e^{\mathbf{x}_i^T\boldsymbol{\beta}_1\left(u_{i^*},v_{i^*}\right)}}\right)\left(1+\eta\left(1-g_1\right)\left(e^{-y_{2i}}-g_2\right)\right)\right)}$

$$
U=\exp\left(\frac{-e^{\mathbf{x}_i^T\boldsymbol{\beta}_1\left(u_{i^*},v_{i^*}\right)}}{1+\varphi_1 e^{\mathbf{x}_i^T\boldsymbol{\beta}_1\left(u_{i^*},v_{i^*}\right)}}-\frac{e^{\mathbf{x}_i^T\boldsymbol{\beta}_2\left(u_{i^*},v_{i^*}\right)}}{1+\varphi_2 e^{\mathbf{x}_i^T\boldsymbol{\beta}_2\left(u_{i^*},v_{i^*}\right)}}\right)\left(\frac{-e^{\mathbf{x}_i^T\boldsymbol{\beta}_2\left(u_{i^*},v_{i^*}\right)}\mathbf{x}_i^T}{1+\varphi_2 e^{\mathbf{x}_i^T\boldsymbol{\beta}_2\left(u_{i^*},v_{i^*}\right)}}-\frac{\left(e^{\mathbf{x}_i^T\boldsymbol{\beta}_2\left(u_{i^*},v_{i^*}\right)}\right)^2\varphi_2\mathbf{x}_i^T}{\left(1+\varphi_2 e^{\mathbf{x}_i^T\boldsymbol{\beta}_2\left(u_{i^*},v_{i^*}\right)}\right)^2}\right)\left(1+\eta\left(1-g_1\right)\left(1-g_2\right)\right)\frac{\partial g_2}{\partial \boldsymbol{\beta}_2\left(u_{i^*},v_{i^*}\right)}
$$

$$
\frac{\partial l}{\partial \varphi_1}=\sum_{i=1}^{n} w_{ii^*}\left(1-b_i-c_i-d_i\right)\left(\frac{1}{S}\left(e^{\mathbf{x}_i^T\boldsymbol{\gamma}_2\left(u_{i^*},v_{i^*}\right)}\exp\left(\frac{-e^{\mathbf{x}_i^T\boldsymbol{\beta}_1\left(u_{i^*},v_{i^*}\right)}}{1+\varphi_1 e^{\mathbf{x}_i^T\boldsymbol{\beta}_1\left(u_{i^*},v_{i^*}\right)}}\right)\frac{-\left(e^{\mathbf{x}_i^T\boldsymbol{\beta}_1\left(u_{i^*},v_{i^*}\right)}\right)^2}{\left(1+\varphi_1 e^{\mathbf{x}_i^T\boldsymbol{\beta}_1\left(u_{i^*},v_{i^*}\right)}\right)^2}+\exp\left(\frac{-e^{\mathbf{x}_i^T\boldsymbol{\beta}_1\left(u_{i^*},v_{i^*}\right)}}{1+\varphi_1 e^{\mathbf{x}_i^T\boldsymbol{\beta}_1\left(u_{i^*},v_{i^*}\right)}}-\frac{e^{\mathbf{x}_i^T\boldsymbol{\beta}_2\left(u_{i^*},v_{i^*}\right)}}{1+\varphi_2 e^{\mathbf{x}_i^T\boldsymbol{\beta}_2\left(u_{i^*},v_{i^*}\right)}}\right)\frac{\left(e^{\mathbf{x}_i^T\boldsymbol{\beta}_1\left(u_{i^*},v_{i^*}\right)}\right)^2}{\left(1+\varphi_1 e^{\mathbf{x}_i^T\boldsymbol{\beta}_1\left(u_{i^*},v_{i^*}\right)}\right)^2}\left(1+\eta\left(1-g_1\right)\left(1-g_2\right)\right)\frac{\partial g_1}{\partial \varphi_1}\right)\right)
$$

$$
+\sum_{i=1}^{n} w_{ii^*}b_i\left(\frac{-\exp\left(\frac{e^{\mathbf{x}_i^T\boldsymbol{\beta}_1\left(u_{i^*},v_{i^*}\right)}}{1+\varphi_1 e^{\mathbf{x}_i^T\boldsymbol{\beta}_1\left(u_{i^*},v_{i^*}\right)}}\right)\left(1+\eta\left(1-g_1\right)\left(e^{-y_{2i}}-g_2\right)\right)\left(e^{\mathbf{x}_i^T\boldsymbol{\beta}_1\left(u_{i^*},v_{i^*}\right)}\right)^2}{\left(e^{\mathbf{x}_i^T\boldsymbol{\gamma}_1\left(u_{i^*},v_{i^*}\right)}+\exp\left(\frac{-e^{\mathbf{x}_i^T\boldsymbol{\beta}_1\left(u_{i^*},v_{i^*}\right)}}{1+\varphi_1 e^{\mathbf{x}_i^T\boldsymbol{\beta}_1\left(u_{i^*},v_{i^*}\right)}}\right)\left(1+\eta\left(1-g_1\right)\left(e^{-y_{2i}}-g_2\right)\right)\right)\left(1+\varphi_1 e^{\mathbf{x}_i^T\boldsymbol{\beta}_1\left(u_{i^*},v_{i^*}\right)}\right)^2}\frac{\partial g_1}{\partial \varphi_1}\right)
$$

$$
+\sum_{i=1}^{n} w_{ii^*}c_i\left(y_{1i}\frac{e^{\mathbf{x}_i^T\boldsymbol{\beta}_1\left(u_{i^*},v_{i^*}\right)}}{\left(1+\varphi_1 e^{\mathbf{x}_i^T\boldsymbol{\beta}_1\left(u_{i^*},v_{i^*}\right)}\right)}+\frac{\left(y_{1i}-1\right)}{\left(1+\varphi_1 y_1\right)}y_{1i}+\left(\frac{-e^{\mathbf{x}_i^T\boldsymbol{\beta}_1\left(u_{i^*},v_{i^*}\right)}y_{1i}}{1+\varphi_1 e^{\mathbf{x}_i^T\boldsymbol{\beta}_1\left(u_{i^*},v_{i^*}\right)}}+\frac{\left(e^{\mathbf{x}_i^T\boldsymbol{\beta}_1\left(u_{i^*},v_{i^*}\right)}\right)^2\left(1+\varphi_1 y_1\right)}{\left(1+\varphi_1 e^{\mathbf{x}_i^T\boldsymbol{\beta}_1\left(u_{i^*},v_{i^*}\right)}\right)^2}\right)+\frac{\exp\left(\frac{-e^{\mathbf{x}_i^T\boldsymbol{\beta}_2\left(u_{i^*},v_{i^*}\right)}}{1+\varphi_2 e^{\mathbf{x}_i^T\boldsymbol{\beta}_2\left(u_{i^*},v_{i^*}\right)}}\right)\left(1+\eta\left(1-g_2\right)\left(e^{-y_{1i}}-g_1\right)\right)}{\left(e^{\mathbf{x}_i^T\boldsymbol{\gamma}_2\left(u_{i^*},v_{i^*}\right)}+\exp\left(\frac{-e^{\mathbf{x}_i^T\boldsymbol{\beta}_2\left(u_{i^*},v_{i^*}\right)}}{1+\varphi_2 e^{\mathbf{x}_i^T\boldsymbol{\beta}_2\left(u_{i^*},v_{i^*}\right)}}\right)\left(1+\eta\left(1-g_2\right)\left(e^{-y_{1i}}-g_1\right)\right)\right)}\frac{\partial g_1}{\partial \varphi_1}\right)
$$

$$
+\sum_{i=1}^{n} w_{ii^*}d_i\left(\frac{-y_{1i}e^{\mathbf{x}_i^T\boldsymbol{\beta}_1\left(u_{i^*},v_{i^*}\right)}}{1+\varphi_1 e^{\mathbf{x}_i^T\boldsymbol{\beta}_1\left(u_{i^*},v_{i^*}\right)}}+\frac{\left(y_{1i}-1\right)y_{1i}}{\left(1+\varphi_1 y_{1i}\right)}+\left(\frac{-e^{\mathbf{x}_i^T\boldsymbol{\beta}_1\left(u_{i^*},v_{i^*}\right)}}{1+\varphi_1 e^{\mathbf{x}_i^T\boldsymbol{\beta}_1\left(u_{i^*},v_{i^*}\right)}}\left(y_{1i}-\frac{e^{\mathbf{x}_i^T\boldsymbol{\beta}_1\left(u_{i^*},v_{i^*}\right)}\left(1+\varphi_1 y_{1i}\right)}{\left(1+\varphi_1 e^{\mathbf{x}_i^T\boldsymbol{\beta}_1\left(u_{i^*},v_{i^*}\right)}\right)}\right)\right)+\frac{1}{\left(1+\eta\left(e^{-y_{1i}}-g_1\right)\left(e^{-y_{2i}}-g_2\right)\right)}\frac{\partial g_1}{\partial \varphi_1}\right)
$$

$$\frac{\partial l}{\partial \varphi_2}=\sum_{i=1}^{n} w_{ii^*}\left(1-b_i-c_i-d_i\right)\left(\frac{1}{S}\left(e^{\mathbf{x}_i^T\boldsymbol{\gamma}_1\left(u_{i^*},v_{i^*}\right)}\exp\left(\frac{-e^{\mathbf{x}_i^T\boldsymbol{\beta}_2\left(u_{i^*},v_{i^*}\right)}}{1+\varphi_2 e^{\mathbf{x}_i^T\boldsymbol{\beta}_2\left(u_{i^*},v_{i^*}\right)}}\right)\frac{-\left(e^{\mathbf{x}_i^T\boldsymbol{\beta}_2\left(u_{i^*},v_{i^*}\right)}\right)^2}{\left(1+\varphi_2 e^{\mathbf{x}_i^T\boldsymbol{\beta}_2\left(u_{i^*},v_{i^*}\right)}\right)^2}+\exp\left(\frac{-e^{\mathbf{x}_i^T\boldsymbol{\beta}_2\left(u_{i^*},v_{i^*}\right)}}{1+\varphi_2 e^{\mathbf{x}_i^T\boldsymbol{\beta}_2\left(u_{i^*},v_{i^*}\right)}}-\frac{e^{\mathbf{x}_i^T\boldsymbol{\beta}_1\left(u_{i^*},v_{i^*}\right)}}{1+\varphi_1 e^{\mathbf{x}_i^T\boldsymbol{\beta}_1\left(u_{i^*},v_{i^*}\right)}}\right)\frac{\left(e^{\mathbf{x}_i^T\boldsymbol{\beta}_2\left(u_{i^*},v_{i^*}\right)}\right)^2}{\left(1+\varphi_2 e^{\mathbf{x}_i^T\boldsymbol{\beta}_2\left(u_{i^*},v_{i^*}\right)}\right)^2}K\frac{\partial g_2}{\partial \varphi_2}\right)\right)$$

$$+\sum_{i=1}^{n} w_{ii^*}b_i\left(-y_{2i}\frac{e^{\mathbf{x}_i^T\boldsymbol{\beta}_2\left(u_{i^*},v_{i^*}\right)}}{\left(1+\varphi_2 e^{\mathbf{x}_i^T\boldsymbol{\beta}_2\left(u_{i^*},v_{i^*}\right)}\right)}+\frac{\left(y_{2i}-1\right)}{\left(1+\varphi_2 y_2\right)}y_{2i}+\left(\frac{-e^{\mathbf{x}_i^T\boldsymbol{\beta}_2\left(u_{i^*},v_{i^*}\right)}y_{2i}}{1+\varphi_2 e^{\mathbf{x}_i^T\boldsymbol{\beta}_2\left(u_{i^*},v_{i^*}\right)}}+\frac{(e^{\mathbf{x}_i^T\boldsymbol{\beta}_2\left(u_{i^*},v_{i^*}\right)})^2\left(1+\varphi_2 y_2\right)}{\left(1+\varphi_2 e^{\mathbf{x}_i^T\boldsymbol{\beta}_2\left(u_{i^*},v_{i^*}\right)}\right)^2}\right)+\frac{\exp\left(\frac{-e^{\mathbf{x}_i^T\boldsymbol{\beta}_1\left(u_{i^*},v_{i^*}\right)}}{1+\varphi_1 e^{\mathbf{x}_i^T\boldsymbol{\beta}_1\left(u_{i^*},v_{i^*}\right)}}\right)K}{\left(e^{\mathbf{x}_i^T\boldsymbol{\gamma}_1\left(u_{i^*},v_{i^*}\right)}+\exp\left(\frac{-e^{\mathbf{x}_i^T\boldsymbol{\beta}_1\left(u_{i^*},v_{i^*}\right)}}{1+\varphi_1 e^{\mathbf{x}_i^T\boldsymbol{\beta}_1\left(u_{i^*},v_{i^*}\right)}}\right)K\right)}\frac{\partial g_2}{\partial \varphi_2}\right)$$

$$+\sum_{i=1}^{n} w_{ii^*}c_i\left(\frac{-\exp\left(\frac{e^{\mathbf{x}_i^T\boldsymbol{\beta}_2\left(u_{i^*},v_{i^*}\right)}}{1+\varphi_2 e^{\mathbf{x}_i^T\boldsymbol{\beta}_2\left(u_{i^*},v_{i^*}\right)}}\right)\left(1+\eta\left(1-g_2\right)\left(e^{-y_{1i}}-g_1\right)\right)\left(e^{\mathbf{x}_i^T\boldsymbol{\beta}_2\left(u_{i^*},v_{i^*}\right)}\right)^2}{\left(e^{\mathbf{x}_i^T\boldsymbol{\gamma}_2\left(u_{i^*},v_{i^*}\right)}+\exp\left(\frac{-e^{\mathbf{x}_i^T\boldsymbol{\beta}_2\left(u_{i^*},v_{i^*}\right)}}{1+\varphi_2 e^{\mathbf{x}_i^T\boldsymbol{\beta}_2\left(u_{i^*},v_{i^*}\right)}}\right)\left(1+\eta\left(1-g_2\right)\left(e^{-y_{1i}}-g_1\right)\right)\right)\left(1+\varphi_2 e^{\mathbf{x}_i^T\boldsymbol{\beta}_2\left(u_{i^*},v_{i^*}\right)}\right)^2}\frac{\partial g_2}{\partial \varphi_2}\right)$$

$$+\sum_{i=1}^{n} w_{ii^*}d_i\left(\frac{-y_{2i}e^{\mathbf{x}_i^T\boldsymbol{\beta}_2\left(u_{i^*},v_{i^*}\right)}}{1+\varphi_2 e^{\mathbf{x}_i^T\boldsymbol{\beta}_2\left(u_{i^*},v_{i^*}\right)}}+\frac{\left(y_{2i}-1\right)y_{2i}}{\left(1+\varphi_2 y_{2i}\right)}+\left(\frac{-e^{\mathbf{x}_i^T\boldsymbol{\beta}_2\left(u_{i^*},v_{i^*}\right)}}{1+\varphi_2 e^{\mathbf{x}_i^T\boldsymbol{\beta}_2\left(u_{i^*},v_{i^*}\right)}}\left(y_{2i}-\frac{e^{\mathbf{x}_i^T\boldsymbol{\beta}_2\left(u_{i^*},v_{i^*}\right)}\left(1+\varphi_2 y_{2i}\right)}{\left(1+\varphi_2 e^{\mathbf{x}_i^T\boldsymbol{\beta}_2\left(u_{i^*},v_{i^*}\right)}\right)}\right)\right)+\frac{1}{\left(1+\eta\left(e^{-y_{1i}}-g_1\right)\left(e^{-y_{2i}}-g_2\right)\right)}\frac{\partial g_2}{\partial \varphi_2}\right)$$

where $K=\left(1+\eta\left(1-g_1\right)\left(e^{-y_{2i}}-g_2\right)\right)$

$$\frac{\partial l}{\partial \eta}=\sum_{i=1}^{n} w_{ii^*}\left(1-b_i-c_i-d_i\right)\left(\frac{1}{S}\left(\exp\left(-\frac{e^{\mathbf{x}_i^T\boldsymbol{\beta}_1\left(u_{i^*},v_{i^*}\right)}}{1+\varphi_1 e^{\mathbf{x}_i^T\boldsymbol{\beta}_1\left(u_{i^*},v_{i^*}\right)}}-\frac{e^{\mathbf{x}_i^T\boldsymbol{\beta}_2\left(u_{i^*},v_{i^*}\right)}}{1+\varphi_2 e^{\mathbf{x}_i^T\boldsymbol{\beta}_2\left(u_{i^*},v_{i^*}\right)}}\right)\left(1-g_1\right)\left(1-g_2\right)\right)\right)$$

$$+\sum_{i=1}^{n} w_{ii^*}b_i\left(\frac{\exp\left(\frac{-e^{\mathbf{x}_i^T\boldsymbol{\beta}_1\left(u_{i^*},v_{i^*}\right)}}{1+\varphi_1 e^{\mathbf{x}_i^T\boldsymbol{\beta}_1\left(u_{i^*},v_{i^*}\right)}}\right)\left(1-g_1\right)\left(e^{-y_{2i}}-g_2\right)}{\left(e^{\mathbf{x}_i^T\boldsymbol{\gamma}_1\left(u_{i^*},v_{i^*}\right)}+\exp\left(\frac{-e^{\mathbf{x}_i^T\boldsymbol{\beta}_1\left(u_{i^*},v_{i^*}\right)}}{1+\varphi_1 e^{\mathbf{x}_i^T\boldsymbol{\beta}_1\left(u_{i^*},v_{i^*}\right)}}\right)\left(1+\eta\left(1-g_1\right)\left(e^{-y_{2i}}-g_2\right)\right)\right)}\right)$$

$$+\sum_{i=1}^{n} w_{ii^*}c_i\left(\frac{\exp\left(\frac{-e^{\mathbf{x}_i^T\boldsymbol{\beta}_2\left(u_{i^*},v_{i^*}\right)}}{1+\varphi_2 e^{\mathbf{x}_i^T\boldsymbol{\beta}_2\left(u_{i^*},v_{i^*}\right)}}\right)\left(1-g_2\right)\left(e^{-y_{1i}}-g_1\right)}{\left(e^{\mathbf{x}_i^T\boldsymbol{\gamma}_2\left(u_{i^*},v_{i^*}\right)}+\exp\left(\frac{-e^{\mathbf{x}_i^T\boldsymbol{\beta}_2\left(u_{i^*},v_{i^*}\right)}}{1+\varphi_2 e^{\mathbf{x}_i^T\boldsymbol{\beta}_2\left(u_{i^*},v_{i^*}\right)}}\right)\left(1+\eta\left(1-g_2\right)\left(e^{-y_{1i}}-g_1\right)\right)\right)}\right)+\sum_{i=1}^{n} w_{ii^*}d_i\left(\frac{\left(e^{-y_{1i}}-g_1\right)\left(e^{-y_{2i}}-g_2\right)}{1+\eta\left(e^{-y_{1i}}-g_1\right)\left(e^{-y_{2i}}-g_2\right)}\right)$$

Appendix 2: Log likelihood under population (GWBZIGPR)

$$\log A_{4i}^{*}=\log\frac{1}{1+e^{\mathbf{x}_i^T\hat{\boldsymbol{\gamma}}_1(u_i,v_i)}}\frac{1}{1+e^{\mathbf{x}_i^T\hat{\boldsymbol{\gamma}}_2(u_i,v_i)}}+\log\left(e^{\mathbf{x}_i^T\hat{\boldsymbol{\gamma}}_1(u_i,v_i)}e^{\mathbf{x}_i^T\hat{\boldsymbol{\gamma}}_2(u_i,v_i)}+e^{\mathbf{x}_i^T\hat{\boldsymbol{\gamma}}_1(u_i,v_i)}\exp\left(\frac{-e^{\mathbf{x}_i^T\hat{\boldsymbol{\beta}}_2(u_i,v_i)}}{1+\varphi_2e^{\mathbf{x}_i^T\hat{\boldsymbol{\beta}}_2(u_i,v_i)}}\right)+e^{\mathbf{x}_i^T\hat{\boldsymbol{\gamma}}_2(u_i,v_i)}\exp\left(\frac{-e^{\mathbf{x}_i^T\hat{\boldsymbol{\beta}}_1(u_i,v_i)}}{1+\varphi_1e^{\mathbf{x}_i^T\hat{\boldsymbol{\beta}}_1(u_i,v_i)}}\right)+A_1\right)$$

where $A_1=\exp\left(\frac{-e^{\mathbf{x}_i^T\hat{\boldsymbol{\beta}}_1(u_i,v_i)}}{1+\varphi_1e^{\mathbf{x}_i^T\hat{\boldsymbol{\beta}}_1(u_i,v_i)}}-\frac{e^{\mathbf{x}_i^T\hat{\boldsymbol{\beta}}_2(u_i,v_i)}}{1+\varphi_2e^{\mathbf{x}_i^T\hat{\boldsymbol{\beta}}_2(u_i,v_i)}}\right)\left(1+\eta(1-g_1)(1-g_2)\right)$

$$\log B_{4i}^{*}=\log\frac{1}{1+e^{\mathbf{x}_i^T\hat{\boldsymbol{\gamma}}_2(u_i,v_i)}}+y_{2i}\mathbf{x}_i^T\hat{\boldsymbol{\beta}}_2(u_i,v_i)-y_{2i}\log\left(1+\hat{\varphi}_2e^{\mathbf{x}_i^T\hat{\boldsymbol{\beta}}_2(u_i,v_i)}\right)+(y_{2i}-1)\log(1+\hat{\varphi}_2y_{2i})-\log y_{2i}!+\left(\frac{-e^{\mathbf{x}_i^T\hat{\boldsymbol{\beta}}_2(u_i,v_i)}(1+\hat{\varphi}_2y_{2i})}{1+\hat{\varphi}_2e^{\mathbf{x}_i^T\hat{\boldsymbol{\beta}}_2(u_i,v_i)}}\right)+B_1$$

where $B_1=\log\frac{1}{1+e^{\mathbf{x}_i^T\hat{\boldsymbol{\gamma}}_1(u_i,v_i)}}+\log\left(e^{\mathbf{x}_i^T\hat{\boldsymbol{\gamma}}_1(u_i,v_i)}+\exp\left(\frac{-e^{\mathbf{x}_i^T\hat{\boldsymbol{\beta}}_1(u_i,v_i)}}{1+\hat{\varphi}_1e^{\mathbf{x}_i^T\hat{\boldsymbol{\beta}}_1(u_i,v_i)}}\right)\left(1+\eta(1-g_1)\left(e^{-y_{2i}}-g_2\right)\right)\right)$

$$\log C_{4i}^{*}=\log\frac{1}{1+e^{\mathbf{x}_i^T\hat{\boldsymbol{\gamma}}_1(u_i,v_i)}}+y_{1i}\log e^{\mathbf{x}_i^T\hat{\boldsymbol{\beta}}_1(u_i,v_i)}-y_{1i}\log\left(1+\varphi_1e^{\mathbf{x}_i^T\hat{\boldsymbol{\beta}}_1(u_i,v_i)}\right)+(y_{1i}-1)\log(1+\hat{\varphi}_1y_{1i})-\log y_{1i}!+\left(\frac{-e^{\mathbf{x}_i^T\hat{\boldsymbol{\beta}}_1(u_i,v_i)}(1+\hat{\varphi}_1y_{1i})}{1+\hat{\varphi}_1e^{\mathbf{x}_i^T\hat{\boldsymbol{\beta}}_1(u_i,v_i)}}\right)+\log C_1+\log C_2$$

where $C_1=\frac{1}{1+e^{\mathbf{x}_i^T\hat{\boldsymbol{\gamma}}_2(u_i,v_i)}}$ and $C_2=\left(e^{\mathbf{x}_i^T\hat{\boldsymbol{\gamma}}_2(u_i,v_i)}+\exp\left(\frac{-e^{\mathbf{x}_i^T\hat{\boldsymbol{\beta}}_2(u_i,v_i)}}{1+\hat{\varphi}_2e^{\mathbf{x}_i^T\hat{\boldsymbol{\beta}}_2(u_i,v_i)}}\right)\left(1+\eta(1-g_2)\left(e^{-y_{1i}}-g_1\right)\right)\right)$

$$\log D_{4i}^{*}=\log\frac{1}{1+e^{\mathbf{x}_i^T\hat{\boldsymbol{\gamma}}_1(u_i,v_i)}}+\log\frac{1}{1+e^{\mathbf{x}_i^T\hat{\boldsymbol{\gamma}}_2(u_i,v_i)}}+y_{1i}\mathbf{x}_i^T\hat{\boldsymbol{\beta}}_1(u_i,v_i)-y_{1i}\log\left(1+\hat{\varphi}_1e^{\mathbf{x}_i^T\hat{\boldsymbol{\beta}}_1(u_i,v_i)}\right)+(y_{1i}-1)\log(1+\hat{\varphi}_1y_{1i})-\log y_{1i}!+\left(\frac{-e^{\mathbf{x}_i^T\hat{\boldsymbol{\beta}}_1(u_i,v_i)}(1+\hat{\varphi}_1y_{1i})}{1+\hat{\varphi}_1e^{\mathbf{x}_i^T\hat{\boldsymbol{\beta}}_1(u_i,v_i)}}\right)+D_1$$

$$D_1=y_{2i}\mathbf{x}_i^T\boldsymbol{\beta}_2(u_i,v_i)-y_{2i}\log\left(1+\hat{\varphi}_2e^{\mathbf{x}_i^T\hat{\boldsymbol{\beta}}_2(u_i,v_i)}\right)+(y_{2i}-1)\log(1+\hat{\varphi}_2y_{2i})-\log y_{2i}!+\left(\frac{-e^{\mathbf{x}_i^T\hat{\boldsymbol{\beta}}_2(u_i,v_i)}(1+\hat{\varphi}_2y_{2i})}{1+\hat{\varphi}_2e^{\mathbf{x}_i^T\hat{\boldsymbol{\beta}}_2(u_i,v_i)}}\right)+\log\left(1+\eta\left(e^{-y_{1i}}-g_1\right)\left(e^{-y_{2i}}-g_2\right)\right)$$

Appendix 3: Log likelihood under H_0 (GWBZIGPR)

$$\log A_{7i}=\log\frac{1}{1+e^{\mathbf{x}_i^T\boldsymbol{\gamma}_1(u_{i^*},v_{i^*})}}\frac{1}{1+e^{\mathbf{x}_i^T\boldsymbol{\gamma}_2(u_{i^*},v_{i^*})}}+\ln\left(e^{\mathbf{x}_i^T\boldsymbol{\gamma}_1(u_{i^*},v_{i^*})}e^{\mathbf{x}_i^T\boldsymbol{\gamma}_2(u_{i^*},v_{i^*})}+e^{\mathbf{x}_i^T\boldsymbol{\gamma}_1(u_{i^*},v_{i^*})}\exp\left(\frac{-e^{\beta_{20}(u_{i^*},v_{i^*})}}{1+\varphi_2e^{\beta_{20}(u_{i^*},v_{i^*})}}\right)+e^{\mathbf{x}_i^T\boldsymbol{\gamma}_2(u_{i^*},v_{i^*})}\exp\left(\frac{-e^{\beta_{10}(u_{i^*},v_{i^*})}}{1+\varphi_1e^{\beta_{10}(u_{i^*},v_{i^*})}}\right)+A_1\right)$$

where $A_1=\exp\left(\frac{-e^{\beta_{10}(u_{i^*},v_{i^*})}}{1+\varphi_1e^{\beta_{10}(u_{i^*},v_{i^*})}}-\frac{e^{\beta_{20}(u_{i^*},v_{i^*})}}{1+\varphi_2e^{\beta_{20}(u_{i^*},v_{i^*})}}\right)\left(1+\eta(1-g_1)(1-g_2)\right)$

$$\log B_{7i}=\log\frac{1}{1+e^{\mathbf{x}_i^T\boldsymbol{\gamma}_2(u_{i^*},v_{i^*})}}+y_{2i}\beta_{20}(u_{i^*},v_{i^*})-y_{2i}\log\left(1+\varphi_2e^{\beta_{20}(u_{i^*},v_{i^*})}\right)+(y_{2i}-1)\log(1+\varphi_2y_{2i})-\log y_{2i}!+\left(\frac{-e^{\beta_{20}(u_{i^*},v_{i^*})}(1+\varphi_2y_{2i})}{1+\varphi_2e^{\beta_{20}(u_{i^*},v_{i^*})}}\right)+B_1$$

where $B_1=\log\frac{1}{1+e^{\mathbf{x}_i^T\boldsymbol{\gamma}_1(u_{i^*},v_{i^*})}}+\log\left(e^{\mathbf{x}_i^T\boldsymbol{\gamma}_1(u_{i^*},v_{i^*})}+\exp\left(\frac{-e^{\beta_{10}(u_{i^*},v_{i^*})}}{1+\varphi_1e^{\beta_{10}(u_{i^*},v_{i^*})}}\right)\left(1+\eta(1-g_1)\left(e^{-y_{2i}}-g_2\right)\right)\right)$

$$\log C_{1i}=\log\frac{1}{1+e^{\mathbf{x}_i^T\boldsymbol{\gamma}_1(u_{i^*},v_{i^*})}}+y_{1i}\log e^{\beta_{10}(u_{i^*},v_{i^*})}-y_{1i}\log\left(1+\varphi_1e^{\beta_{10}(u_{i^*},v_{i^*})}\right)+(y_{1i}-1)\log(1+\varphi_1y_{1i})-\log y_{1i}!+C_1+\log C_2+\log C_3$$

where $C_1=\left(\frac{-e^{\beta_{10}(u_{i^*},v_{i^*})}(1+\varphi_1y_{1i})}{1+\varphi_1e^{\beta_{10}(u_{i^*},v_{i^*})}}\right)$, $C_2=\frac{1}{1+e^{\mathbf{x}_i^T\boldsymbol{\gamma}_2(u_{i^*},v_{i^*})}}$ and $C_3=\left(e^{\mathbf{x}_i^T\boldsymbol{\gamma}_2(u_{i^*},v_{i^*})}+\exp\left(\frac{-e^{\beta_{20}(u_{i^*},v_{i^*})}}{1+\varphi_2e^{\beta_{20}(u_{i^*},v_{i^*})}}\right)\left(1+\eta(1-g_2)\left(e^{-y_{1i}}-g_1\right)\right)\right)$

$$\log D_{1i}=\log\frac{1}{1+e^{\mathbf{x}_i^T\boldsymbol{\gamma}_1(u_{i^*},v_{i^*})}}+\log\frac{1}{1+e^{\mathbf{x}_i^T\boldsymbol{\gamma}_2(u_{i^*},v_{i^*})}}+y_{1i}\beta_{10}(u_{i^*},v_{i^*})-y_{1i}\log\left(1+\varphi_1e^{\beta_{10}(u_{i^*},v_{i^*})}\right)+(y_{1i}-1)\log(1+\varphi_1y_{1i})-\log y_{1i}!+\left(\frac{-e^{\beta_{10}(u_{i^*},v_{i^*})}(1+\varphi_1y_{1i})}{1+\varphi_1e^{\beta_{10}(u_{i^*},v_{i^*})}}\right)+D_1$$

where

$$D_1=y_{2i}\beta_{20}(u_{i^*},v_{i^*})-y_{2i}\log\left(1+\varphi_2e^{\beta_{20}(u_{i^*},v_{i^*})}\right)+(y_{2i}-1)\log(1+\varphi_2y_{2i})-\log y_{2i}!+\left(\frac{-e^{\beta_{20}(u_{i^*},v_{i^*})}(1+\varphi_2y_{2i})}{1+\varphi_2e^{\beta_{20}(u_{i^*},v_{i^*})}}\right)+\log\left(1+\eta\left(e^{-y_{1i}}-g_1\right)\left(e^{-y_{2i}}-g_2\right)\right)$$

Appendix 4: Sub-District Grouping Based on Significant Variables in the Zero State Model

Sub-District		Variable
**The Number of Pregnant Maternal Mortality**	**The Number of Postpartum Maternal Mortality**	
Salem, Banjarharjo	Salem	X_4
	Bantarkawung, Ketanggungan	X_1, X_5
Larangan		X_1, X_2, X_3
Bantarkawung, Ketanggungan, Tanjung		X_1, X_3, X_4
	Tanjung	X_1, X_3, X_5
	Margasari, Bumiayu, Jatibarang, Larangan, Kersana, Bulukamba, Wanasari, Brebes, Songgom	X_1, X_2, X_3, X_5
	Reban, Bawang, Tersono, Gringsing, Limpung, Banyuputih, Batang, Wonokerto, Pulosari, Bumijawa, Bojong, Balapulang, Pagerbarang, Lebaksiu, Jatinegara, Kedungbanteng, Pangkah, Slawi, Dukuhwaru, Adiwerna, Dukuhturi, Talang, Tarub, Suradadi, Paguyangan, Sirampog, Tonjong, Pekalongan Barat, Pekalongan Timur, Pekalongan Utara, Tegal Selatan, Tegal timur, Tegal Barat, Margadana	X_1, X_2, X_3, X_5, X_6
Margasari, Bumiayu, Jatibarang, Kersana, Bulukamba, Wanasari, Brebes, Songgom		X_1, X_2, X_3, X_4, X_5
Bumijawa, Balapulang, Pagerbarang, Slawi, Dukuhwaru, Adiwerna, Dukuhturi, Paguyangan, Sirampog, Tonjong, Tegal Selatan, Tegal Timur, Tegal Barat, Margadana	Wonotunggal, Bandar, Blado, Subah, Pencalungan, Tulis, Kandeman, Warungasem, Lebakbarang, Petungkriyono, Talun, Kramat	X_1, X_2, X_3, X_4, X_5, X_6
	Banjarharjo, Losari	Nothing significant
	Kandangserang, Paninggaran, Doro, Karanganyar, Kajen, Kesesi, Sragi, Siwalan, Bojong, Wonopringgo, Kedungwuni, Karangdadap, Buaran, Tirto, Wiradesa, Moga, Warungpring, Belik, Watukumpul, Bodeh, Bantarbolang, Randudongkal, Pemalang, Taman, Petarukan, Ampelgading, Comal, Ulujami, Warureja, Pekalongan Selatan	X_1, X_2, X_3, X_5, X_6, X_7
Wonotunggal, Bandar, Blado, Reban, Bawang, Tersono, Gringsing, Limpung, Banyuputih, Subah, Pecalungan, Tulis, Kandeman, Batang, Warungasem, Kandangserang, Paninggaran, Lebakbarang, Petungkriyono, Talun, Doro, Karanganyar, Kajen, Kesesi, Sragi, Siwalan, Bojong, Wonopringgo, Kedungwuni, Karangdadap, Buaran, Tirto, Wiradesa, Wonokerto, Moga, Warungpring, Pulosari, Belik, Watukumpul, Bodeh, Bantarbolang, Randudongkal, Pemalang, Taman, Petarukan, Ampelgading, Comal, Ulujami, Bojong, Lebaksiu, Jatinegara, Kedungbanteng, Pangkah, Talang, Tarub, Kramat, Suradadi, Warureja, Pekalongan Barat, Pekalongan Timur, Pekalongan Utara, Pekalongan Selatan		X_1, X_2, X_3, X_4, X_5, X_6, X_7

Appendix 5: Sub-District Grouping Based on Significant Variables in the Poisson State Model

Sub-District		Variable
**The Number of Pregnant Maternal Mortality**	**The Number of Postpartum Maternal Mortality**	
	Salem, Banjarharjo, Losari	X_1, X_3
	Bantarkawung, Ketanggungan, Tanjung, Bulukamba	X_1, X_2, X_3,
Losari		X_1, X_2, X_3, X_4
	Wanasari	X_1, X_2, X_3, X_5
Salem		X_1, X_2, X_3, X_6
Larangan, Ketanggungan, Banjarharjo, Tanjung, Kersana, Bulukamba, Wanasari		X_1, X_2, X_3, X_4, X_6
	Margasari, Bumiayu, Jatibarang, Larangan, Kersana, Brebes, Songgom	X_1, X_2, X_3, X_5, X_6
	Bumijawa, Balapulang, Pagerbarang, Slawi, Dukuhwaru, Adiwerna, Dukuhturi, Paguyangan, Sirampog, Tonjong, Tegal Selatan, tegal Timur, Tegal Barat, Margadana	X_1, X_2, X_3, X_4, $X_{5,}$, X_6
Margasari, Bumijawa, Balapulang, Pagebarang, Slawi, Dukuhwaru, Adiwerna, Dukuhturi, Bumiayu, Paguyangan, Sirampog, Tonjong, Jatibarang, Brebes, Tegal Selatan, Tegal Barat, Tegal Timur, margadana		X_1, X_2, X_3, X_4, $X_{6,}$, X_7
Songgom		X_1, X_2, X_3, X_5, X_6, X_7
Wonotunggal, Bandar, Blado, Reban, Bawang, Tersono, Gringsing, Limpung, Banyuputih, Subah, Pecalungan, Tulis, Kandeman, Batang, Warungasem, Kandangserang, Paninggaran, Lebakbarang, Petungkriyono, Talun, Doro, Karanganyar, Kajen, Kesesi, Sragi, Siwalan, Bojong, Wonopringgo, Kedungwuni, Karangdadap, Buaran, Tirto, Wiradesa, Wonokerto, Moga, Warungpring, Pulosari, Belik, Watukumpul, Bodeh, Bantarbolang, Randudongkal, Pemalang, Taman, Petarukan, Ampelgading, Comal, Ulujami, Bojong, Lebaksiu, Jatinegara, Kedungbanteng, Pangkah, Talang, Tarub, Kramat, Suradadi, Warureja, Pekalongan Barat, Pekalongan Timur, Pekalongan Utara, Pekalongan Selatan	Wonotunggal, Bandar, Blado, Reban, Bawang, Tersono, Gringsing, Limpung, Banyuputih, Subah, Pecalungan, Tulis, Kandeman, Batang, Warungasem, Kandangserang, Paninggaran, Lebakbarang, Petungkriyono, Talun, Doro, Karanganyar, Kajen, Kesesi, Sragi, Siwalan, Bojong, Wonopringgo, Kedungwuni, Karangdadap, Buaran, Tirto, Wiradesa, Wonokerto, Moga, Warungpring, Pulosari, Belik, Watukumpul, Bodeh, Bantarbolang, Randudongkal, Pemalang, Taman, Petarukan, Ampelgading, Comal, Ulujami, Bojong, Lebaksiu, Jatinegara, Kedungbanteng, Pangkah, Talang, Tarub, Kramat, Suradadi, Warureja, Pekalongan Barat, Pekalongan Timur, Pekalongan Utara, Pekalongan Selatan	X_1, X_2, X_3, X_4, X_5, X_6, X_7
